# The Role of Epigenetics in Corneal Fibrosis

**DOI:** 10.3390/epigenomes10020040

**Published:** 2026-06-06

**Authors:** Julia T. Coelho, Ella J. Dewald, Syeda R. Ali, Moira L. Geary, Mithun Santra, Gary H. F. Yam

**Affiliations:** 1 Department of Ophthalmology, University of Pittsburgh, Pittsburgh, PA 15219, USA; jtc91@pitt.edu (J.T.C.); ejd96@pitt.edu (E.J.D.); sra160@pitt.edu (S.R.A.); mlo39@pitt.edu (M.L.G.); mithun.santra@pitt.edu (M.S.); 2 McGowan Institute for Regenerative Medicine, University of Pittsburgh, Pittsburgh, PA 15219, USA

**Keywords:** epigenetics, corneal fibrosis, DNA methylation, histone modification, non-coding RNAs, biomarkers, treatment

## Abstract

Epigenetics regulates gene activity without altering the underlying DNA sequences. Numerous studies have highlighted the importance of epigenetics in diverse physiological processes, including cell growth, differentiation, and tissue development. Increasingly, epigenetic modifications are recognized for their involvement in various diseases, notably corneal disorders. Corneal fibrosis, a common consequence of ocular injury or infection, significantly contributes to visual impairment and blindness worldwide. Recent evidence indicates that epigenetic changes regulate key processes in corneal pathogenesis, such as inflammation, wound healing, extracellular matrix remodeling, fibrosis, and neovascularization. These findings underscore the potential of developing novel therapeutic strategies that specifically target epigenetic mechanisms to treat or mitigate corneal pathology. Nevertheless, bringing epigenetic therapies into clinical practice remains challenging given the complexity of epigenetic regulation. Future research leveraging multi-omics technologies and specific gene manipulation will be essential to elucidate the mechanisms underlying epigenetic regulation in corneal diseases and to identify specific therapeutic targets. Such advancements will drive the development of effective, clinically relevant treatments for corneal fibrosis and related disorders.

## 1. Introduction

A clear cornea is essential for normal vision, as it transmits light without interference. The cornea provides about two-thirds of the eye’s refractive power, focusing light onto the retina, and acts as a robust barrier protecting internal ocular tissues. The central corneal region is avascular and consists of three cellular layers: the outermost corneal epithelium (CEpi), middle corneal stroma (CSt), and innermost corneal endothelium (CEndo). These layers are arranged in a lamellar structure, separated by two acellular interfaces: Bowman’s membrane between the superficial CEpi and anterior CSt, and Descemet’s membrane (DM) between the posterior CSt and CEndo [1].

The stratified squamous CEpi serves as a barrier against microbes, toxins, and fluid penetration, playing a crucial role in protecting the eye from infection while maintaining corneal transparency. The epithelium is continuously regenerated by limbal epithelial stem cells, supporting both normal renewal and rapid injury repair [2,3]. The CSt constitutes approximately 90% of the overall cornea’s volume and is primarily responsible for mechanical strength and optical clarity. It consists of parallel, regularly arranged lamellae rich in collagen fibrils and proteoglycans organized in an orthogonal pattern. This stromal organization enables efficient light transmission with minimal scattering. Resident stromal keratocytes are typically quiescent and function to synthesize collagens and proteoglycans to maintain the stromal extracellular matrix (ECM) [4]. The CEndo, lining the posterior corneal surface, is vital for maintaining corneal transparency by regulating stromal hydration. It achieves this through a balance of passive leakage and active ion-pumping, which control water and solute movement between the CSt and aqueous humor. Dysfunction or loss of corneal endothelial cells disrupts this balance, resulting in stromal edema, collagen fibril irregularity, and ultimately, loss of corneal clarity.

Corneal fibrosis, clinically referred to as corneal scarring, results from dysregulated wound healing that disrupts corneal tissue homeostasis and results in abnormal ECM organization inside the CSt [5]. Corneal opacification or scarring is the fourth leading cause of blindness worldwide, impacting over 12 million individuals (Eye Bank Association of America; https://restoresight.org/cornea-donation/; accessed on 30 March 2026), and representing approximately 3.2% of global blindness cases [6]. Each year, an estimated two million new cases occur [7]. Corneal fibrosis arises from diverse etiologies, including infections (bacterial, viral, and fungal), trauma (mechanical, chemical, thermal), autoimmune disorders (e.g., Sjogren’s syndrome and Stevens–Johnson syndrome), corneal dystrophies (e.g., keratoconus, ectasia, and Fuchs’ endothelial dystrophy), and post-surgical complications (e.g., phototherapeutic keratectomy, keratoplasty, and laser sub-epithelial keratomileusis) [8].

Following corneal stromal injury, damaged keratocytes undergo apoptosis, while surviving keratocytes become activated and transit to proliferating and migratory stromal fibroblasts, which subsequently transform into contractile, α-smooth muscle actin (αSMA) positive myofibroblasts (myoFs). This process is driven by pro-fibrotic signaling involving transforming growth factor-β (TGFβ), platelet-derived growth factor (PDGF), basic fibroblast growth factor (bFGF), and vascular endothelial growth factor (VEGF), as well as inflammatory cytokines including interleukins, interferon-γ (IFNγ), and tumor necrosis factor-α (TNFα) [9,10]. These mediators activate both Smad-dependent and non-Smad pathways, including c-Jun N-terminal kinase (JNK), Janus kinase/signal transducer and activator of transcription (JAK-STAT), and p38 mitogen-activated protein kinase (MAPK). Ultimately, these signaling cascades promote myoF differentiation and persistence, leading to excessive production of disorganized ECM components such as collagen and fibronectin. The resulting disruption of stromal architecture increases light scattering, thereby decreasing corneal transparency and causing visual impairment [11].

Traditional treatment of corneal fibrosis relies primarily on corticosteroids or mitomycin C, which are generally effective for mild to moderate opacities [12]. However, these treatments have side effects and are less effective once the CEpi has healed and stromal opacity has formed, limiting their use largely to prophylactic applications [13,14]. As a result, established corneal scarring often remains refractory to medical management. For conditions of severe fibrosis and opacification, corneal transplantation is the standard treatment. However, this procedure has various restrictions and risks, including infection, wound leakage, graft rejection, infiltrate formation, epithelial ingrowth, postoperative astigmatism, and disease recurrence [15]. Besides the invasive nature and prolonged recovery, corneal transplantation offers modest long-term success, with graft survival rates of approximately 50% at 15 years [16].

In recent years, emerging strategies have been explored for the management of corneal fibrosis. Losartan, an angiotensin II receptor blocker commonly used for systemic hypertension, has shown promise as an off-label topical treatment for corneal fibrosis due to its ability to inhibit TGFβ signaling and suppress myoF formation [17,18]. Unlike standard therapies, losartan penetrates CEpi and the basement membrane, enabling it to reduce fibrosis across various etiologies. However, further studies are needed to define the optimal dosing and assess the long-term efficacy. In addition to pharmacological approaches, stem cell–based treatments have gained increasing interest because of their regenerative potential. Corneal stromal stem cells (CSSCs), for example, modulate corneal inflammation and fibrosis. Preclinical research has shown that topical application of human CSSCs to wound sites suppresses inflammation and fibrosis in animal models of corneal injury, including mechanical, chemical, cryo-injury, and laser-induced damage [19,20,21,22,23,24,25]. The anti-scarring and stromal regenerative effects are largely mediated by extracellular vesicles and exosomes, which deliver anti-fibrotic cytokines and microRNAs [26,27,28]. In addition, non-corneal stem cells and novel technologies such as 3D-printed biomaterials are being investigated to further enhance corneal regeneration and anti-fibrotic outcomes [29,30,31].

Multiple studies have demonstrated that epigenetic mechanisms are involved in the initiation and development of fibrosis. Modulating epigenetic regulation has emerged as a potential strategy for managing fibrosis, such as in cancers, neurological diseases, and autoimmune conditions [32,33,34,35]. Epigenetics refers to heritable or reversible changes in gene expression that occur without altering the underlying DNA sequence. These mechanisms aid in understanding how environmental cues and cellular changes influence gene expression, which controls and regulates phenotypes.

**Figure 1:**
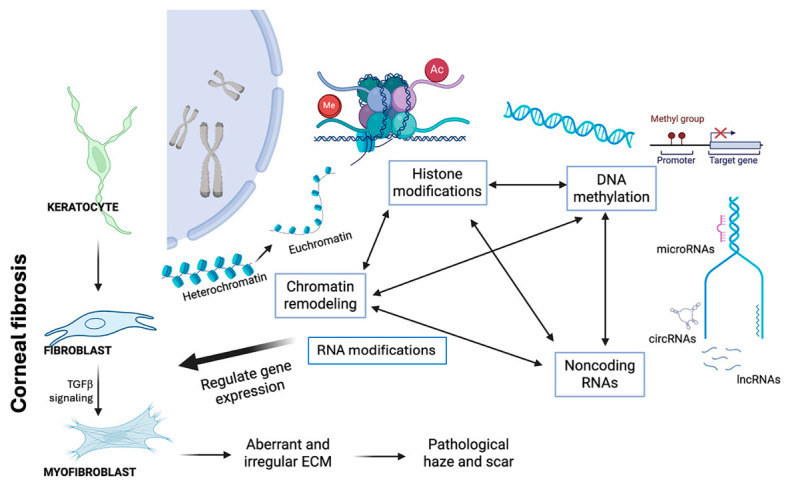
A schematic diagram illustrating the key epigenetic regulatory pathways—chromatin remodeling, histone modification, DNA methylation, and noncoding RNAs—in corneal stromal fibrosis that involves the activation of stromal keratocytes to fibroblasts and myofibroblasts, resulting in aberrant extracellular matrix (ECM) production and ultimately causing corneal opacity and scarring. The figure was created in Biorender.com. Yam G. (2026). https://BioRender.com/gejn3n7 (accessed on 30 April 2026).

Gene expression is a complex multi-step process involving DNA unwinding, transcription, RNA processing and splicing, translation, and post-translational modifications. Each stage can be tightly regulated through epigenetic mechanisms (Figure 1). Chromatin remodeling dynamically modifies chromatin architecture via ATP-dependent complexes, e.g., SWI/SNF, that switch between transcriptionally inactive “closed” heterochromatin and transcriptionally active “open” euchromatin states, hence controlling the accessibility of gene structure to transcriptional machinery [36]. Histone modifications, such as acetylation and methylation, alter DNA-histone interactions and influence chromatin compaction. DNA methylation, typically at the 5th carbon position of cytosine residues within CpG dinucleotides, can obstruct transcription factor access to the DNA major groove [37]. Overall, these mechanisms regulate the accessibility of DNA to transcriptional factors and RNA polymerases, thereby modulating gene transcription. In addition, non-coding RNAs, including microRNAs (miRNAs), circular RNAs (circRNAs), and long non-coding RNAs (lncRNAs), are important regulators of gene expression by interacting with transcription factors and epigenetic modifiers to adjust transcriptional and post-transcriptional processes [38]. Through the coordinated activity of these mechanisms, epigenetic regulation influences each stage of gene expression, resulting in changes to protein expression, function, and overall phenotypes.

In this review, we present an overview of epigenetics, describe its involvement in corneal wound healing and fibrosis, and discuss potential epigenetic targets and modifiers for developing treatments for corneal fibrosis and scarring.

## 2. DNA Methylation in Corneal Fibrosis

DNA methylation is a key epigenetic modification that plays crucial roles in gene expression, cellular differentiation, and organ development. It operates through mechanisms such as transcriptional silencing, X-chromosome inactivation, and genomic imprinting. The methylation pattern reflects a dynamic balance between methylation and demethylation [39], regulated by three classes of enzymes: “writers” or methyltransferases that catalyze DNA methylation; “erasers” or demethylases that remove methylation, and “readers” (methylation-dependent binding proteins) that recognize epigenetic marks [40].

DNA methylation involves the transfer of a methyl group from reactive compounds, e.g., S-adenosylmethionine (SAM), to the 5th carbon position of cytosine residues within CpG (cytosine-guanine) dinucleotides [41]. This event chemically modifies methyl groups with covalent bonding to cytosine residues, forming 5-methylcytosine (5mC) [42]. In response to various factors such as heat stress, nutrition, or infection, 5mC can be oxidized by 5mC oxidase (the ten-eleven translocation TET protein) to 5-hydroxymethylcytosine (5hmC). Since DNA methyltransferases poorly recognize 5hmC, methylation can be lost at 5hmC sites during DNA replication [43]. Many CpG sites are located within gene promoter regions, where they cluster to form CpG islands (CGIs). Methylation of these promoter regions generally leads to transcriptional repression. When a gene is actively expressed, CpG sites within promoter CGIs are usually demethylated, allowing transcription factors to bind and initiate transcription. In contrast, methylated cytosines alter DNA configuration and impede transcription factor binding, thereby reducing transcriptional activity. Additionally, RNA methylation regulates transcriptional expression [44]. Among different forms, m6A with methylation of the nitrogen (N) atom at position 6 of adenine is the most abundant RNA modification in mammals [45]. A study by Hu et al. (2020) identified m6A modifications in 1137 mRNA species (780 hypermethylated and 357 hypomethylated) in mouse corneas following *Fusarium* infection [46].

DNA methylation is mediated by a family of DNA methyltransferases (DNMTs), including DNMT1, DNMT3a, DNMT3b, and DNMT3L [47]. DNMT1 maintains CpG methylation during DNA replication by transferring existing methylation patterns onto newly synthesized DNA strands. DNMT3a and 3b function as de novo methyltransferases that establish new methylation marks on unmethylated DNA, particularly in CGIs. Although DNMT3L lacks catalytic activity, it interacts with DNMT3a and 3b to form complexes that regulate the catalytic activity and enhance overall methylation efficiency.

In corneal fibrosis, aberrant DNA methylation is associated with persistent activation of fibroblasts and myoFs, which are the primary effector cells for excessive ECM production. MyoFs express contractile proteins such as αSMA and produce excessive amounts of collagen and ECM proteins. Epigenetic silencing of anti-fibrotic genes through promoter hypermethylation has been reported in various fibrotic diseases. For example, the anti-fibrotic gene *RASAL1* is hypermethylated in renal fibroblasts, leading to sustained fibroblast activation and progression of kidney fibrosis [48]. These findings demonstrate how stable epigenetic changes can drive chronic fibrotic responses.

DNA methylation plays an important regulatory role in pro-fibrotic signaling. Among the key pathways, TGFβ is recognized as a central regulator of fibrogenesis, controlling fibroblast activation, myoF differentiation, and ECM production. Epigenetic modifications can modulate the expression of components within the TGFβ pathway and its downstream target genes [49]. Abnormal methylation patterns affecting TGFβ signaling may enhance pathway activation, leading to increased fibrotic gene expression. Additionally, DNA methylation-mediated repression of anti-fibrotic regulators can amplify TGFβ-driven fibrotic responses. Conversely, DNA demethylation, regulated by demethylases like the TET enzyme, counterbalances these effects. For example, in a mouse corneal injury model caused by alkali burn, rapamycin treatment reduced mTOR gene promoter methylation, facilitating transcriptional factor binding and upregulating PI3K/AKT/mTOR signaling pathway involved in corneal angiogenesis [50].

Beyond fibroblast and myoF, DNA methylation also influences the behavior of other cell types involved in fibrosis, including epithelial, endothelial, and immune cells. Epigenetic changes in macrophages and inflammatory cells can alter cytokine production and inflammatory responses, thereby modulating the fibrotic environment [51]. DNA methylation is further involved in epithelial-to-mesenchymal transition (EpiMT)**,** a process leading to the expansion of fibrogenic cell populations [52]. In the corneal endothelium, Fuchs’ Endothelial Corneal Dystrophy (FECD) is characterized by progressive CEndo cell apoptosis and degeneration, abnormal ECM deposition on DM (“guttae” formation), and RNA toxicity from *TCF4* gene mutations [53]. These changes disrupt barrier and pump function, resulting in corneal edema, stromal keratocyte death, and fibrosis. DNA methylation array has identified over 10,000 differentially methylated sites in FECD samples compared to controls, with 59% hypermethylated and 41% hypomethylated. These changes dysregulate genes involved in ion transport/pump activity, metabolic processes, and cytoskeletal organization [54]. Additionally, Pan et al. reported aberrant DNA methylation in microRNA gene promoters in FECD patients. Specifically, miR-199b is extensively hypermethylated in FECD, leading to complete silencing of miR-199-5p and loss of its inhibition of the transcription factors Snai1 and ZEB1, which promotes ECM gene expression [55].

In summary, DNA methylation is an important regulator of gene expression programs underlying fibrosis. It influences fibroblast activation, modulates signaling pathways like TGFβ, and interacts with other epigenetic regulators. Continued research into epigenetic mechanisms will deepen our understanding of fibrosis progression and may offer new avenues for therapeutic intervention.

### Pharmacological Regulation of DNA Methylation as a Potential Therapeutic Strategy for Corneal Fibrosis

Unlike genetic mutations, epigenetic modifications are potentially reversible, making them attractive targets for therapeutic intervention. Altered DNA methylation has been reported across multiple fibrotic diseases [56,57]. Silencing of anti-fibrotic genes and activation of pro-fibrotic pathways promote myoF activity and resistance to apoptosis [58]. Notably, DNMT inhibitors such as 5-aza-2′-deoxycytidine (5-Aza-dC) or decitabine have been shown to reverse aberrant methylation and reduce fibrotic gene expression in experimental models of cancers and fibrosis, including breast and colorectal cancer, lymphoma, and liver and endometrial fibrosis [59,60,61,62]. In ocular fibrosis, pretreatment of human conjunctival fibroblasts with 5-Aza-dC before TGFβ2 treatment substantially reduced αSMA and type I collagen expression [63]. Although the mechanisms underlying ECM gene downregulation after 5-Aza-dC treatment remain not fully defined and may differ by cell type and experimental conditions [61], the effect of demethylation can lead to hypomethylation of anti-fibrotic genes such as *RASAL1* and *PTEN*, as reported in liver cirrhosis models [64].

As 5-Aza-dC is approved by the U.S. Food and Drug Administration (FDA) for the treatment of myelodysplastic syndromes (MDS) and acute myeloid leukemia (AML) [65], its established clinical safety and tolerance suggest it could be a good candidate for fibrosis therapies in ocular tissues, including the cornea.

## 3. Post-Translational Histone Modifications

In eukaryotic cells, DNA is packaged into chromatin, with nucleosomes serving as the basic structural units. Each nucleosome comprises approximately 146 to 147 base pairs of DNA wrapped around a histone octamer, which includes two H2A–H2B dimers and one H3–H4 tetramer [66]. The N- and C-terminal tails of histone proteins undergo diverse post-translational modifications, such as acetylation, phosphorylation, methylation, SUMOylation, deamination, ADP-ribosylation, and ubiquitination, dynamically regulated by specific enzymes [67]. Histone acetyltransferases (HATs) catalyze the addition of acetyl groups, causing chromatin relaxation and transcriptional activation. Conversely, histone deacetylases (HDACs) remove acetyl groups, generally repressing gene expression. Histone methyltransferases (HMTs) transfer methyl groups from SAM to lysine or arginine residues on histones H3 and H4, while histone demethylases (HDMs) remove these methyl groups [68,69]. In addition, histone ubiquitination, catalyzed by ubiquitin ligases, modulates chromatin architecture. Acetylation and deacetylation alter the electrostatic charge of histones, affecting their binding affinity for DNA and hence influencing chromatin compactness and organization. Overall, histone acetylation promotes open chromatin and active transcription, whereas deacetylation leads to chromatin condensation and gene repression.

In humans, eighteen HDACs have been identified and are classified into four classes (Table 1) [70]. Several of these HDACs are implicated in organ fibrosis: HDAC2 contributes to liver fibrosis through hepatic stellate cell activation; HDAC3, 8, and 9 are involved in pulmonary fibrosis by promoting fibroblast activation and myoF contraction; and HDAC3 and 6 participate in cardiac fibrosis via TGFβ-Smad2/3 signaling [71,72,73].

Histone modifications are involved in several ophthalmic diseases, including cataracts, posterior capsule opacification, granular corneal dystrophy, and aberrant corneal wound healing [74,75,76]. After corneal injury or surgery, altered histone modification patterns can dysregulate key fibrotic signaling pathways, such as TGFβ, leading to keratocyte activation and differentiation into myoFs, excessive ECM deposition, and ultimately corneal fibrosis. TGFβ signaling regulates histone modifications, such as methylation, acetylation, and phosphorylation, to control the expression of fibrosis-related genes [77]. For instance, TGFβ promotes the recruitment of histone-modifying enzymes like HDACs and HMTs, which regulate genes including COL1A1 and αSMA [78]. These epigenetic changes create a chromatin environment that favors pro-fibrotic expression and suppresses anti-fibrotic pathways. Granular corneal dystrophy type 2 (GCD2, Avellino corneal dystrophy) is impacted by mutations in the TGFβ-induced (TGFBI) gene that encodes the ECM protein TGFBIp. Corneal fibroblasts treated with TGFβ1 showed upregulated expression of TGFBIp and ECM-associated genes such as connective tissue growth factor, collagen-α2[I], and plasminogen activator inhibitor-1. These changes were associated with increased activating histone marks (H3K4me1/3) and reduced repressive marks (H3K27me3), thereby facilitating transcription factor binding and gene activation. Notably, these effects are attenuated in GCD2-derived corneal fibroblasts [75]. Collectively, these findings elucidate the interplay between TGFβ signaling and histone modifications, which have the potential to uncover novel therapeutic strategies to mitigate fibrosis and confer protective effects in disorders such as corneal fibrosis and GCD2.

### Pharmacological Regulation of Histone Modification as a Therapeutic Strategy for Corneal Fibrosis

HDAC inhibitors (HDACis) are small molecules that inhibit zinc-dependent HDAC activity. Several HDACis, including Vorinostat, Givinostat, Abexinostat, Belinostat, Panobinostat, and Trichostatin A, are under investigation in both pre-clinical and clinical settings [79,80].

Trichostatin A (TSA) is a reversible inhibitor of class I and II HDACs that suppresses TGFβ1-induced collagen synthesis, αSMA expression, and myoF differentiation through hyperacetylation of H3 and H4 histones [81,82]. In corneal fibroblasts, TSA downregulates TGFβ1-induced αSMA and fibronectin RNA levels by 60–75%, with a 1.5 to 3-fold decrease in protein expression. Topical application of TSA suppresses corneal haze development in a rabbit model following photorefractive keratectomy [83]. Despite these beneficial effects, the clinical utility of TSA is restricted by its cytotoxicity, including the induction of cell cycle arrest and apoptosis. Suberoylanilide Hydroxamic Acid (SAHA; Vorinostat), a pan-HDAC inhibitor targeting class I, II, and IV HDACs, is FDA-approved for the treatment of cutaneous T-cell lymphoma [84]. In canine corneal fibroblasts, SAHA attenuates TGFβ1-induced Smad2/3 phosphorylation and downstream expression of fibrosis-associated genes [85]. In diabetic mouse models, SAHA enhances corneal epithelial wound healing by upregulating aquaporin-3 (AQP3), which regulates cell hydration, proliferation, and immune responses, and reduces pro-inflammatory cytokines such as IL-1β and TNFα [86].

ITF2357 (Givinostat) exhibits anti-fibrotic effects in both human corneal fibroblasts and rabbit corneal injury models [87]. It inhibits TGFβ signaling by reducing Smad2 phosphorylation and preventing Smad4 association with TGFβ receptor type I kinase, while upregulating bone morphogenic protein 7 (BMP7), a known antagonist of TGFβ signaling. Moreover, sodium butyrate (NaB), a short-chain fatty acid HDAC inhibitor, promotes dedifferentiation of corneal myoFs into fibroblasts and decreases the expression of αSMA, fibronectin, and collagen III [88]. Functionally, NaB reduces collagen gel contractility in vitro and diminishes corneal opacity in vivo after alkali injury.

## 4. Chromatin Remodeling

Chromatin structure undergoes dynamic changes that regulate DNA accessibility and gene expression. Chromatin remodeling is mediated by ATP-dependent chromatin remodeling complexes, such as SWI/SNF, and covalent histone modifications including acetylation, methylation, phosphorylation, and ubiquitination. These processes modulate chromatin compaction, facilitating transitions between transcriptionally active euchromatin and repressive heterochromatin states, thereby controlling gene activity [89]. The major families of chromatin remodelers include SWI/SNF (switch/sucrose-non-fermenting), ISWI (imitation switch), INO80 (inositol requiring 80), and CHD (chromodomain-helicase-DNA binding) [90]. These remodeling complexes have been implicated in various ocular features and pathologies, including wound healing responses, cataract formation, iris pigmentation, and albinism. For instance, mice lacking Brahma (*BRM*), an ATPase subunit of the SWI/SNF complex, develop corneal epithelial hyperplasia and deregulated stromal cell growth following ultraviolet radiation-induced damage [91]. Additionally, mutations in ATPase subunits of ISWI and SWI/SNF complexes disrupt lens development and are associated with cataract formation [92,93]. The helicase-like transcription factor (HLTF), a member of the SWI/SNF family, also regulates iris pigmentation by targeting the *OCA2* gene and modulating melanin production in melanocytes [94].

In the context of fibrosis, chromatin remodeling alters gene accessibility, promoting myoF activation. Altered expression of *BRG1*, a core ATPase subunit of the SWI/SNF complex, has been detected in human cirrhotic liver biopsies, and targeted deletion of *BRG1* in myoFs reduces liver fibrosis in vivo [95]. Under stress conditions, chromatin remodeling modulates immune cell-fibroblast crosstalk, influencing fibroblast activation and fibrotic progression, as shown in heart failure models. Recent epigenomic studies further reveal the role of chromatin accessibility dynamics in corneal fibrosis. ATAC-seq-based analyses have identified extensive chromatin remodeling in fibrotic corneas following alkali injury in rabbits, characterized by increased chromatin condensation, spatial domain segregation, and a shift in regulatory architecture from promoter-proximal to distal regions [96]. For instance, naïve corneas show higher promoter-transcription start site accessibility (~15%), whereas alkali-injured fibrotic corneas exhibit a marked reduction to less than 5%, indicating a loss of promoter-enriched accessibility and redistribution of regulatory elements. These findings demonstrate chromatin-level alterations in gene regulation that control fibrosis development and stromal tissue responses. Despite these advances, evidence in corneal fibrosis remains limited, with substantial knowledge gaps in understanding the mechanistic links between chromatin remodeling and fibrotic gene regulation. This situation underscores the need for further research, as systematic identification and characterization of chromatin accessibility patterns and epigenetic signatures may uncover novel therapeutic targets for the treatment of corneal fibrosis.

## 5. Non-Coding RNAs

Non-coding RNAs (ncRNAs) represent a diverse group of RNA molecules that do not encode proteins but play essential roles in regulating gene expression at transcriptional, post-transcriptional, and epigenetic levels [97]. The major subclasses include microRNAs (miRNAs), long non-coding RNAs (lncRNAs), and circular RNAs (circRNAs). In fibrotic disorders, ncRNAs have emerged as important regulators of fibroblast activation, ECM remodeling, inflammatory signaling, and wound healing outcomes. Through the regulation of signaling pathways such as TGFβ, chromatin-associated events, and RNA regulation, ncRNAs contribute to maintaining the balance between regenerative repair and pathologic scar formation.

### 5.1. MicroRNAs (miRNAs)

MicroRNAs are short, single-stranded RNAs of ~20–25 nucleotides generated from hairpin precursors. They repress gene expression by guiding the RNA-induced silencing complex (RISC) to target specific mRNAs, resulting in translational inhibition or mRNA degradation [98]. Ocular tissues exhibit distinctive miRNA expression patterns; for instance, miR-184 and 205 are highly enriched in cornea and lens, miR-204 in lens epithelial cells, and miR204/211 in retinal pigment epithelium. Dysregulated expression of miRNAs has been linked to different ocular pathologies, including retinal degeneration, angiogenesis, and uveitis, indicating their regulatory roles in eye homeostasis and disease pathogenesis [99,100,101,102].

In corneal fibrosis, miR-21 is consistently upregulated, promoting fibroblast activation and ECM production partly through enhanced TGFβ signaling, while the miR-29 family acts as an anti-fibrotic regulator by directly targeting collagen and ECM-associated genes [27,103]. A similar observation was made in FECD that miRNA profiling identified 87 miRNAs downregulated in FECD samples compared to normal CEndo cells, including miR-29 family members and *DICER1*, which is involved in miRNA biogenesis [104]. The miR-29 family is regarded as a master fibro-regulator influencing ECM homeostasis. Studies have shown that miR-29b suppresses collagen expression in human tenon fibroblasts while the miR-29 family inhibits fibrotic ECM proteins, such as SPARC, collagen I, and IV [105]. More examples of miRNAs in association with ocular fibrosis are listed in Table 2. In addition to their roles in tissue biology, miRNAs are being explored as biomarkers in ocular surface disease. Tear miRNA profiling has identified distinct expression signatures in vernal keratoconjunctivitis, while altered profiles in neurotrophic keratitis have implicated miR-146a and miR-424 in delayed wound healing [106,107].

Notably, miRNAs appear to mediate part of the therapeutic effect of extracellular vesicles (EVs). EVs derived from human corneal stromal stem cells (hCSSCs) reduce corneal fibrosis and scarring in murine injury models, and depletion of EV-associated miRNAs diminishes this anti-scarring effect, supporting a functional potential of miRNA cargo in tissue repair [26]. Elevated miR-29a expression in hCSSC-derived EVs further supports the concept in delivering anti-inflammatory and anti-fibrotic effects through miRNA-mediated paracrine signaling [27,108]. Since EV-associated miRNAs are relatively stable and selectively packaged, they hold promise as minimally invasive tear-based biomarkers for monitoring corneal injury and fibrosis.

**Table 2:** Examples of miRNAs in association with tissue fibrosis.

miRNA	Potential Targets	Reported Functions	Literatures
miR-26a	PTEN-PI3K/AKT signaling, Smad4, connective tissue growth factor CTGF, EZH2	Acts as epithelial–mesenchymal transition (EpiMT) suppressors in fibrotic and inflammatory reactions	[109,110,111,112,113,114,115]
miR-26b	PDGFRβ, HMGA2, CTGF, Smad4, COX2		
miR-145	KLF4, FGF10 ZEB2, SOX9	Promotes fibroblast-to-myofibroblast differentiation Prevents fibrosis via targeting NFκB, AKT/GSK-3β/β-catenin signaling	[116,117,118]
miR-29 family	Collagens, ECM genes, DNMTs, multiple targets	Attenuates fibrosis via MCJ inhibition and Hippo signaling	[27,119,120]
miR-381	KLF6, ANO1	Liver fibrosis	[121,122]
miR-143	Sprouty3, TAK1, CCL20	Cardiac, renal, and liver fibrosis	[123,124,125]
miR-215	Mcm10, Cdc25A, BMPR2	Inhibits fibroblast cell cycling and proliferation	[126,127]
miR-361	NFκB p65	Hepatic stellate cell fibrogenesis; corneal fibrosis	[128,129]

### 5.2. Long Non-Coding RNAs (lncRNAs)

LncRNAs are generally defined as non-coding transcripts longer than 200 nucleotides. They exert diverse molecular functions based on their localization and interacting partners. Within the nucleus, lncRNAs recruit chromatin modifiers and transcriptional regulators to target gene loci, while in the cytoplasm, they act as molecular decoys, scaffolds, competing endogenous RNAs, or modulators of mRNA stability and translation [130]. LncRNAs are classified by their genomic positions relative to neighboring protein-coding genes, and they may be antisense, intronic, divergent, intergenic, promoter-associated, or transcription start site-associated.

More than 100,000 lncRNAs have been annotated in human tissues, and a growing number are implicated in fibrotic diseases. These lncRNAs act as molecular switches controlling fibroblast proliferation, differentiation into myoFs, and ECM deposition [131]. For instance, H19, MEG3, GAS5, NEAT1, and lnc-LFAR1 regulate hepatic stellate cell proliferation and apoptosis in liver fibrosis [132]. Wisper, MHRT, and CHAST are fibroblast-enriched lncRNAs that control cardiac remodeling [133], while PFAR functions as a sponge for miR-138 to promote lung fibroblast activation [134]. In the cornea, lncRNAs participate in both fibrotic and inflammatory responses. LINC00963 interacts with miR-143-3p and modulates TGFβ1-induced αSMA expression in corneal fibroblasts, implicating its role in stromal cell transdifferentiation and myoF formation [135]. In addition, NEAT1 (nuclear enriched abundant transcript 1) is upregulated in lipopolysaccharide-stimulated human corneal fibroblasts and may contribute to corneal inflammation and neovascularization via its interaction with miR-1246 [136]. While the role of lncRNAs in corneal fibrosis remains less well-defined, evidence from other fibrotic systems supports their potential involvement in regulating inflammatory signaling and fibrotic remodeling.

### 5.3. Circular RNAs (circRNAs)

CircRNAs are endogenous, covalently closed RNA molecules generated through back-splicing, which confers high resistance to exonuclease degradation and remarkable stability. Despite being generally expressed at low abundance, circRNAs regulate gene expression through several mechanisms, most notably by acting as miRNA sequestration (“sponge”). Through these interactions, circRNAs influence cell proliferation, migration, apoptosis, angiogenesis, and processes associated with tissue remodeling and fibrosis across multiple organ systems [137]. CircRNAs are broadly classified into exonic circRNAs, exon-intron circRNAs, and circular intronic RNAs [138].

In the cornea, most evidence for circRNA function comes from studies focused on corneal neovascularization (CNV). RNA profiling has revealed numerous dysregulated circRNAs in CNV models. For instance, in an alkali burn-induced mouse CNV model, over 200 circRNAs were differentially expressed between normal and vascularized corneas. Among them, circKIFAP3 suppressed endothelial cell proliferation, migration, and tube formation, suggesting a protective role against pathological NV [139]. In addition, circZNF609 acts as a sponge for miR-184, modulating corneal epithelial cell proliferation, migration, and NV, potentially via the AKT/β-catenin/VEGF signaling axis [140]. Given that corneal fibrosis and NV often co-occur after severe injuries such as chemical burns, infection, or trauma, it is plausible that circRNA-mediated regulation affects both processes. However, direct evidence linking circRNAs to corneal fibrosis remains limited, with much of the mechanistic characterization inferred from other fibrotic tissues. This gap underscores the need for further research to clarify cornea-specific circRNA functions, elucidate underlying mechanisms, and determine whether circRNA-targeted strategies may benefit corneal fibrosis.

Overall, ncRNAs are important molecules regulating corneal fibrosis, with miRNAs, lncRNAs, and circRNAs influencing fibroblast activity, inflammatory responses, ECM deposition, and angiogenic remodeling.

### 5.4. ncRNA-Based Therapeutic Strategy for Corneal Fibrosis

miRNA-based therapeutics hold potential in mitigating corneal fibrosis by modulating key molecular pathways involved in myoF differentiation and inflammation. The fibrotic pathway is primarily driven by TGFβ signaling, which promotes keratocyte-to-myoF transition and excessive collagen synthesis. miRNAs regulate this process post-transcriptionally by targeting multiple components of the fibrotic cascades. Among these, miR-29 family members are particularly characterized by their ability to suppress collagen and various ECM genes. Delivery of miR-29 via EVs has been shown to reduce stromal scarring and inflammation in a mouse corneal injury model [27]. In contrast, profibrotic miRNAs such as miR-21 and miR-145 are upregulated following injury and enhance fibrosis by promoting TGFβ/Smad signaling and αSMA expression. miR-21 amplifies fibrotic signaling through the suppression of SMAD7, while miR-145 regulates myoF differentiation via inhibition of KLF4 [141,142,143]. Inhibition of these miRNAs using antagomirs has been shown to reduce myoF formation, ECM deposition, and corneal opacity in experimental models [106,144]. Furthermore, in a rabbit model of anterior stromal ablation by keratectomy, treatment with lipid nanoparticle (LNP)-encapsulated miRNA-29b significantly reduced fibrosis development, downregulated fibrotic gene expression, such as COL1A2, COL3A1, Fn, and αSMA, to levels similar as naïve corneas, and promoted epithelial healing, stromal structural recovery, and corneal transparency, offering a promising strategy to reduce corneal scarring [145]. Alternatively, treatment with docosahexaenoic acid (DHA) and ε-polylysine (ε-PL) self-assembled miR-361–3p complexes exhibits potent bacteriostatic effects and inhibits corneal fibrosis by disrupting crosstalk between corneal stromal cells and myoF in a bacterial keratitis mouse model [129]. On the other hand, the protective lncRNA LINC00963 inhibits miR-143-3p expression, which is known to increase myoF activity. Thus, blocking this miRNA could suppress the progression of corneal fibrosis [135].

Therapeutically, miRNAs can be delivered as synthetic mimics or inhibitors, or through EV-based systems that provide enhanced stability, biocompatibility, and efficient cellular uptake. Mechanistically, miRNA-based therapies converge on key fibrotic pathways, including the suppression of Smad2/3 activation, the reduction of ECM gene expression, and the modulation of inflammatory cell infiltration. Although challenges remain in optimizing delivery efficiency, minimizing off-target effects, and translating preclinical findings into clinical practice, miRNA therapeutics represent a powerful multi-target approach to promote scarless corneal healing and improve visual outcomes.

## 6. Epigenetic Crosstalk

Epigenetic crosstalk describes the dynamic interplay among histone modifications, DNA methylation, chromatin remodeling, and ncRNAs. These mechanisms do not act independently; instead, they form an integrated network in which individual epigenetic marks can recruit, reinforce, or antagonize one another to establish specific chromatin states [146]. For instance, post-translational modification of histones, such as H3K9me, recruits DNMTs, which promote DNA methylation and induce transcriptional repression [147]. Conversely, active histone marks, such as histone acetylation, are often associated with reduced DNA methylation and increased chromatin accessibility. Hence, epigenetic crosstalk could influence whether chromatin adopts an open (euchromatic) configuration and is permissive to transcription or a condensed (heterochromatic) state and repressive. Dysregulation of these interactions affecting gene regulation has been implicated in a wide range of pathological conditions, including cancer, fibrosis, and degenerative diseases. Importantly, the epigenome’s high plasticity makes epigenetic regulators attractive therapeutic targets for reversing aberrant gene expression programs.

In fibrosis, epigenetic crosstalk can function as a regulatory network that advocates transcriptional programs driving myoF differentiation and abnormal ECM production. Repressive histone marks, such as H3K9me3 and H3K27me3, recruit DNMTs to silence anti-fibrotic genes. In contrast, histone acetylation mediated by HATs promotes chromatin accessibility and facilitates the activation of profibrotic pathways, including TGFβ signaling [146,148]. Chromatin-modifying agents can also influence DNA methylation indirectly. For instance, Sanders et al. found that treating rat lung fibroblasts with HDAC inhibitor TSA demethylated previously hypermethylated sites in the Thy-1 promoter region [149]. TSA also altered DNMT1 activity in reducing global DNA methylation in cancer cell lines [150]. These findings suggest that HDAC inhibition promotes histone acetylation and chromatin relaxation, indirectly affecting DNA methylation by altering DNMT recruitment or activity and associated chromatin-modifying complexes. This effect establishes an indirect mechanism of epigenetic crosstalk. Concurrently, ncRNAs interact with chromatin-modifying enzymes to modulate epigenetic states, either by recruiting histone modifiers to specific genomic regions or regulating DNMT and HDAC expression [151,152].

A recent study by Zhang et al. identified a potential link of ALYREF/UHRF1/RHOB axis linking DNA methylation and RNA modification in the regulation of corneal epithelial wound healing [153]. UHRF1 (Ubiquitin-like with PHD and Ring Finger Domains 1) functions as a central epigenetic integrator, coordinating DNA methylation and histone modification by recruiting DNMT1 to replication forks [154] and binding to repressive histone marks such as H3K9me3 to maintain heterochromatin [155]. Its expression is post-transcriptionally regulated by the binding of ALYREF (Aly/REF export factor) to m5C sites on UHRF1 mRNA, which enhances its stability and translation. Elevated UHFR1 expression subsequently enhances RHOB (Ras homolog family member B) promoter DNA methylation, leading to its suppression and facilitating corneal epithelial wound healing [153]. This study demonstrates that UHRF1 expression is regulated by RNA m5C modification, and UHRF1 in turn mediates RHOB promoter DNA methylation.

Evidence of epigenetic crosstalk within the cornea is currently restricted to epithelial wound healing, and direct links to corneal stromal fibrosis remain limited. Analogous epigenetic interactions have been implicated in fibroblast activation and fibrotic remodeling in other organ systems, suggesting that similar regulatory pathways may operate in corneal fibrosis, though this has yet to be established. Collectively, the dynamic interactions among epigenetic mechanisms could direct cells to adopt a regenerative or fibrotic phenotype. The plasticity of the epigenome presents a therapeutic opportunity, as targeting key nodes of epigenetic crosstalk, such as DNMTs, HDACs, and ncRNA pathways, may reverse established fibrosis and restore tissue function [156].

## 7. Perspective of Epigenetics in Managing Corneal Fibrosis

Exploring the mechanisms of epigenetic regulation can offer a better understanding of gene control and the complexities of disease pathologies, like corneal fibrosis, which involves activation of keratocytes, their transit into fibroblasts, and myoF, a process primarily driven by TGFβ signaling and sustained by biomechanical cues within the ECM. At the molecular level, fibrosis progression is accompanied by coordinated modulation of pro- and anti-fibrotic genes, as well as dysregulated expression of matrix metalloproteinases and their inhibitors. These transcriptional programs are tightly controlled by epigenetic mechanisms that modulate chromatin accessibility and gene expression. Hence, these areas offer important potential to develop appropriate diagnostic, therapeutic, and preventive tools for managing corneal fibrosis and scarring.

### 7.1. Developing Epigenetics as Biomarkers

In fibrotic corneas, aberrant DNA methylation and histone modification patterns underpin the sustained activation of profibrotic programs. For instance, hypermethylation of anti-fibrotic gene promoters and enrichment of repressive histone marks, such as H3K9me3, support a stable fibrotic phenotype, while increased histone acetylation at profibrotic loci facilitates gene activation. In addition, dysregulated microRNAs, including miR-29 and miR-145, orchestrate ECM remodeling and fibroblast activation, thereby driving fibrosis development [142,145]. Collectively, these findings support the concept that epigenetic alterations represent robust and functionally relevant biomarkers for diagnosing corneal fibrosis.

Importantly, these epigenetic signatures are detectable not only in tissue but also in tear fluid and circulating EVs and exosomes, enabling minimally invasive biomarker assessments for clinical applications [27,157]. Their sensitivity to disease staging and responsiveness to therapeutic intervention further support their value for longitudinal monitoring of disease progression and therapeutic efficacy. Moreover, the reversible nature of epigenetic modifications makes these biomarkers attractive targets for therapeutic intervention. Hence, integrating epigenetic profiling into diagnostic and clinical platforms offers an exciting strategy to advance early detection, patient stratification, and personalized medicine in the management of corneal fibrosis.

Technical advances in epigenetic profiling have revolutionized our understanding of fibrosis. DNA methylation microarrays (methylation chips) enable high-throughput analysis of CpG methylation across the genome [158]. In fibrosis research, these technologies have revealed aberrant methylation patterns associated with myoF differentiation, ECM remodeling, inflammatory signaling, and impaired healing processes [149,159]. For example, Pan et al. identified extensive hypermethylation of the miR-199b promoter in FECD patients, leading to silencing of miR-199-5p and subsequent de-repression of *Snai1* and *ZEB1*, which in turn activate ECM genes [55].

Rapid advances in single-cell and spatial epigenomics are transforming fibrosis research by overcoming the limitations of bulk assays, which obstruct the diversity of epithelial, stromal, endothelial, and immune cell populations. These technologies enable analysis of chromatin accessibility, DNA methylation, and histone modifications at single-cell resolution, linking regulatory dynamics to cell-state transitions and microenvironmental cues. For instance, single-cell RNA sequencing of naïve and alkali-injured rabbit corneas has identified 14 transcriptionally distinct cell clusters, revealing a bifurcating stromal trajectory from quiescent keratocytes to activated fibroblasts and myoFs. This finding highlights the dynamic ECM remodeling and intercellular communication underlying in vivo fibrosis [12]. Complementary ATAC-seq analysis of alkali-injured corneas reveals widespread epigenomic remodeling, including loss of promoter accessibility and enrichment of KLF17, NRF2, and ETV6 motifs, suggesting activation of fibrosis-associated regulatory networks [96]. In the epithelial compartment, single-cell multiome profiling after injury shows rapid convergence of distinct epithelial layers on a shared stress and wound-response program, characterized by reduced differentiation markers, increased migratory features, chromatin remodelling, and enrichment of Fosl1/AP-1 motifs [160]. Notably, only a small subset of injury-induced genes show strong coupling between local chromatin accessibility and gene expression, underscoring the complexity of early repair-associated epigenetic regulation.

Spatially resolved epigenomic approach further preserves tissue architecture while mapping regulatory states across regions [161]. This is particularly relevant for fibrosis arising from different aetiologies that involve wound edges, stromal depths, and matrix niches. These platforms can elucidate how gradients of cytokines, hypoxia, inflammatory signals, and matrix stiffness shape local chromatin landscapes during fibrosis development. In parallel, emerging single-cell DNA methylome technologies are valuable for rare clinical specimens, where locus-specific methylation heterogeneity can influence cell lineage plasticity and tissue repair [162,163].

Collectively, methylation arrays, single-cell, and spatial epigenomic approaches offer the potential to identify rare pathogenic fibroblast or epithelial subsets, construct epigenetic trajectories from homeostasis to fibrosis, and reveal cell-type- and locus-specific regulatory targets for epigenetic intervention in fibrotic disorders.

### 7.2. Epigenetic–Metabolic Coupling in Corneal Fibrosis

Recent studies of fibrotic disorders in the liver, lung, and kidney have identified conserved epigenetic–metabolic pathways, which may also be relevant to corneal fibrotic pathology. In these tissues, fibrotic activation is increasingly attributed to metabolic reprogramming, particularly shifts toward glycolysis and mitochondrial dysfunction, which is closely linked to epigenetic remodeling [164,165,166]. Changes in chromatin structure, histone modifications, and ncRNAs regulate epithelial–mesenchymal plasticity and fibrosis-associated cell state transition, underscoring the presence of shared epigenetic–metabolic circuits [167]. The interplay between metabolism and epigenetic regulation helps stabilize fibrotic cell states, and this offers potential therapeutic entry points. Notably, DNA methylation, histone marks such as H3K27me3, H3K27ac, and H3K4me3, and ncRNA-mediated regulation have been implicated in sustaining profibrotic gene expression in stellate cells and fibroblasts during fibrotic reprogramming [168,169]. In the liver, lncRNA H19–EZH2 complexes promote Wnt/β-catenin-driven stellate cell activation and epithelial–mesenchymal transition through remodeling H3K27me3 landscapes [170]. Meanwhile, hepatocyte lipid-associated profibrotic pathways linked to PPARα interact with epigenetic regulation through chromatin readers and remodelers such as BAZ2B [171]. In the kidney, lactylation, nuclear translocation of PKM2, and DNA/histone modifications promote glycolytic and mitochondrial reprogramming in tubular cells and fibroblasts, establishing feedback loops that sustain fibrosis [172].

Given these conserved fibrotic pathways, it is plausible that a similar epigenetic–metabolic coupling contributes to corneal fibrosis, in which the states of stromal and epithelial cells are tightly regulated by metabolic activity and chromatin dynamics. Recent studies by Jeon et al. showed that inhibiting the mitochondrial pyruvate carrier alters Krebs cycle flux, increases intracellular acetyl-CoA levels, and enhances histone H3 acetylation. This metabolic remodeling suppresses myoF phenotype and reduces corneal fibrosis in vivo [173,174]. Also, in a mouse model of alkali burn, corneal fibrosis was associated with increased m6A RNA methylation and upregulation of methyltransferase-like 3 (METTL3); whereas siRNA-mediated silencing of METTL3 increased heat-shock protein 70 expression and reduced fibrosis [175]. Collectively, these preliminary findings support the concept that metabolic reprogramming and epigenetic remodeling interact in corneal fibrosis. Further studies are required to elucidate the underlying mechanisms and determine whether novel molecules or pathways arising from epigenetic–metabolic interaction could offer therapeutic potential for corneal fibrosis.

### 7.3. Developing Epigenetics as a Therapeutic Tool

Epigenetic therapeutics uniquely modulate gene expression without altering the underlying DNA sequence, offering advantages over permanent gene-editing approaches. By precisely targeting epigenetic mechanisms, these treatments can selectively “turn on” or “turn off” genes involved in disease while preserving genomic integrity [176]. Examples of clinical trials on epigenetic therapies involving inhibitors of DNA demethylation and histone deacetylation, as summarized in Table 3, demonstrate proof-of-concept safety and tolerability for various epigenetic drugs, even though most do not directly target fibrotic disorders or ocular diseases. For instance, the DNMT inhibitor 5-Aza-dC/decitabine is approved for treating hematologic malignancies such as AML and MDS by reactivating silenced tumor suppressor genes [177]. Another notable example is Vorinostat (suberoylanilide hydroxamic acid, SAHA), an HDAC inhibitor approved for cutaneous T-cell lymphoma [84], which reactivates tumor suppressor genes and induces cancer cell cycle arrest or apoptosis [178]. More recently, Sood et al. reported that sodium butyrate (NaB), an HDAC inhibitor, serves as an epigenetic treatment for corneal fibrosis. This agent enhances histone acetylation and suppresses profibrotic gene expression in corneal stromal cells [88]. By modulating chromatin structure, NaB reduces myoF differentiation and aberrant ECM deposition.

A major benefit of epigenetic therapies is their reversibility and the avoidance of permanent changes in DNA sequence. Because epigenetic marks are dynamic, treatment effects can often be modulated or reversed, allowing for more controlled interventions than irreversible gene edits [176]. However, this flexibility also introduces risks, such as off-target effects and unintended global changes in gene expression that may disrupt normal cellular function.

DNA methylation and histone acetylation are fundamental regulatory mechanisms that maintain normal corneal cell physiology, supporting epithelial renewal, keratocyte quiescence, endothelial integrity, and immune homeostasis. Broad inhibition of DNMTs or HDACs can unintentionally disrupt gene expression in healthy cells. For example, 5-Aza-dC irreversibly incorporates into replicating DNA and traps DNMT enzymes, potentially inducing DNA damage responses and widespread transcriptional dysregulation in proliferating epithelial or limbal stem cell populations. This may result in unintended cell cycle arrest or senescence in corneal epithelial progenitors and stromal cells, compromising corneal homeostasis and tissue repair. Likewise, TSA and other HDAC inhibitors affect multiple signaling pathways beyond those related to fibrosis, possibly causing cytotoxicity, impaired epithelial wound closure, mitochondrial stress, or altered immune responses. These nonspecific effects are especially concerning in the cornea, where transparency depends on tightly regulated cellular organization and minimal inflammation. Hence, the therapeutic window between beneficial anti-fibrotic modulation and detrimental suppression of physiological wound healing must be carefully defined.

Although 5-Aza-dC/decitabine and TSA have shown promising anti-fibrotic effects in experimental models, their clinical translation for corneal fibrosis faces several pharmacokinetic and safety challenges. The ocular surface possesses highly efficient protective barriers, including the tear film, densely packed CEpi, tight junctions, and rapid tear turnover, that markedly reduce stromal retention of drugs after topical administration. Hydrophilic compounds like 5-Aza-dC have poor epithelial permeability and are prone to rapid degradation in the tear environment, making it difficult to achieve sustained therapeutic effects in the stroma. Furthermore, frequent blinking and nasolacrimal drainage remove most topically applied drugs within minutes, necessitating frequent dosing or the development of specialized delivery systems such as nanoparticles, hydrogels, liposomes, microneedles, or collagen-based carriers to enhance corneal retention and penetration. Targeted inhibitors that focus on fibrosis-associated epigenetic regulators, rather than globally suppressing DNMTs or HDACs, may reduce toxicity to healthy corneal cells and better preserve normal wound healing.

In summary, while epigenetic therapeutics are promising, several limitations suggest that successful clinical translation for corneal fibrosis will require targeted, temporally controlled delivery strategies rather than nonspecific, prolonged topical exposure. Comprehensive investigations into precise targeting and long-term safety will be essential to advance clinical development.

## 8. Conclusions and Future Directions

Epigenetics has emerged as a crucial regulator of cellular and tissue development, as well as the progression of diverse diseases, including cancers and fibrotic disorders. Recent research reveals the complexity of epigenetic modifications, including DNA methylation, histone modifications, and ncRNAs, in modulating gene expression and driving pathological changes. Despite significant progress, a comprehensive understanding of the epigenetic mechanisms underlying disease progression remains elusive. Critical knowledge gaps exist, particularly regarding the dynamic interaction and crosstalk between genetic and epigenetic factors. Future research should focus on elucidating and mapping the precise epigenetic regulatory networks involved in disease onset and development, leveraging high-resolution epigenomic profiling and functional studies. Rapid advancements in single-cell and spatial epigenomics enable simultaneous measurement of transcripts, chromatin accessibility, DNA methylation, and histone modifications within individual cells, providing unprecedented capacity to connect regulatory element dynamics to fibrotic state transitions and environmental cues [179,180].

In the field of corneal fibrosis and related disorders, high-resolution cell type-specific analyses of epigenetic regulation across various cellular compartments are essential for advancing our understanding of the networks and mechanisms governing homeostasis and disease progression. Developing cell-type-resolved epigenomic atlases that integrate single-cell or spatial multi-omics approaches, such as mapping DNA methylation, histone modification landscapes, chromatin accessibility, and ncRNA profiles during fibrotic progression and resolution, will help identify cell-state-specific regulatory networks and elucidate cell lineage transitions involved in myoF formation, persistence, reversal, or apoptosis.

In parallel, exosome or extracellular vesicle (EV)-mediated epigenetic signaling is emerging as a translationally significant area in fibrosis research. Building on the established anti-fibrotic effects of CSSC-derived exosomes and their miRNA-containing cargo, future research should systemically characterize the epigenetic contents, such as ncRNAs and RNA modifiers, and identify factors that reprogram recipient cells to suppress fibrosis and promote stromal regeneration. In addition, engineering EVs with validated epigenetic cargo could facilitate targeted, cell-free therapeutic strategies.

Another direction involves developing cornea-specific epigenetic biomarkers for clinical applications. Profiling epigenetic signatures in tear samples or circulating EVs could enable non-invasive staging of fibrosis progression and severity, as well as prediction of treatment responses.

Finally, in-depth mechanistic studies are crucial for functionally validating epigenetic regulators, employing targeted overexpression and knockdown strategies in clinically relevant injury models of both acute and chronic scarring. By deepening our understanding of the epigenetic landscape and its role in disease pathophysiology, these efforts could pave the way for personalized interventions that improve patient outcomes, prevent disease progression, and enhance quality of life.

## Figures and Tables

**Table 1:** Classes and members of HDACs.

Class	Members	Catalytic Dependence	Primary Localization
I	HDAC1, 2, 3, 8	Zinc-dependent	Nucleus
II	Subclass IIa—HDAC4, 5, 7, 9	Zinc-dependent	Nucleus & cytoplasm
	Subclass IIb—HDAC6, 10	Zinc-dependent	Cytoplasm
III (Sirtuins)	SIRT1, 2, 3, 4, 5, 6, 7	NAD-dependent	Nucleus & cytoplasm, mitochondria
IV	HDAC11	Zinc-dependent	Cytoplasm

NAD—nicotinamide adenine dinucleotide.

**Table 3:** Examples of clinical trials of epigenetic therapy at ClinicalTrials.gov.

Target	Drug	Indications	Trial	Study Phase	Centre	Year
HDAC inhibitor	Depsipeptide	Cutaneous and peripheral T cell lymphoma OM—No. of patients with response; duration of response; adverse events.	NCT00007345	II	Multi	2001–2015
	Belinostat (PXD101)	Recurrent or refractory cutaneous and peripheral T cell lymphomas OM—response rate; time to progression; duration of response.	NCT00274651 (PXD101-CLN-6)	II	Multi	2006–2009
	Belinostat (PXD101)	Acute myeloid leukemia OM—response rate, overall survival; toxicity.	NCT00357032	II	Single	2006–2010
	Belinostat (PXD101)	Ovarian, peritoneal cancer OM—response rate, time to progression; progression-free survival.	NCT00301756	II	Multi	2006–2012
	Panobinostat (LBH589)	Refractory cutaneous T-cell lymphoma OM—response rate, time to response; duration; progression-free survival.	NCT00425555	II	Multi	2007–2013
	Panobinostat (LBH589)	Relapsed multiple myeloma OM—overall and progression-free survival; response rate.	NCT01023308 (PANORAMA-1)	III	Multi	2009–2015
	Entinostat (SNDX-275)	Relapsed or refractory Hodgkin’s lymphoma OM—response rate; duration of response; adverse events.	NCT00866333 (ENGAGE-501)	II	Multi	2009–2013
	Entinostat (SNDX-275)	Metastatic renal cell cancer OM—response rate; dose-limiting toxicity; progression-free survival.	NCT01038778	I/II	Multi	2009–2027
	Panobinostat (LBH589)	Hodgkin’s lymphoma OM—adverse events.	NCT01034163	III	Multi	2010–2012
	Pracinostat (SB939)	Translocation-associated recurrent/metastatic sarcomas OM—response rate.	NCT01112384 (INC200)	II	Multi	2010–2014
	Belinostat (PXD101)	Stage IV non-small-cell lung cancer OM—maximum tolerated dose; safety; progression-free survival.	NCT01310244	I/II	Multi	2010–2015
	Givinostat	Chronic myeloproliferative neoplasms OM—long-term safety and efficacy; progression-free survival.	NCT01761968	II	Multi	2013–2026
	Exemestane +/− Entinostat	Recurrent hormone receptor-positive breast cancer OM—overall survival; objective response rate; progression-free survival; patient-reported quality of life.	NCT02115282	III	Multi	2014–2026
DNMT inhibitor	Azacitidine	High-risk myelodysplastic syndromes OM—time to death; time to transformation to acute myeloid leukemia.	NCT00071799	III	Multi	2003–2007
	Decitabine	Ovarian cancer OM—maximum tolerated dose; objective response; progression-free survival.	NCT00477386	I/II	Single	2007–2013
HDAC inhibitor and DNMT inhibitor	Pracinostat (SB939) Azacitidine	Myelodysplastic syndrome OM—response rate; progression-free survival; rate of leukemia transformation; adverse event profile.	NCT01873703	II	Multi	2013–2016
	Entinostat Azacitidine	Advanced non-small-cell lung cancer OM—maximum tolerated dose; minimum effective dose; pharmacokinetics; antitumor activity; methylation status.	NCT00387465	I/II	Multi	2006–2014
	Vorinostat Decitabine	Advanced solid tumors or relapsed or refractory non-Hodgkin’s lymphoma OM—maximum tolerated dosage; objective response rate; overall survival.	NCT00275080	I	Multi	2006–2014
	Entinostat Azacitidine	Metastatic colorectal cancer OM—tumor response; time to progression; adverse events.	NCT01105377	II	Multi	2010–2014
	Entinostat (SNDX-275) Azacitidine	Advanced breast cancer OM—response rate; clinical benefit rate; patient survival.	NCT01349959	II	Multi	2011–2023
	Pracinostat (SB939) Azacitidine	Acute myeloid leukemia OM—response rate; cytogenetic response; duration of response; patient survival.	NCT01912274	II	Multi	2013–2016

Note: DNMT—DNA methyltransferase; HDAC—histone deacetylase; OM—outcome measures.

## Data Availability

All data are included in the text. Data details are available from the corresponding author on request.

## Abbreviations

The following abbreviations are used in this manuscript:

5-Aza-dC	5-aza-2′-deoxycytidine
5hmC	5-hydroxymethylcytosine
5mC	5-methylcytosine
AML	Acute myeloid leukemia
AQP3	Aquaporin-3
bFGF	Basic fibroblast growth factor
BMP7	Bone morphogenic protein 7
CEndo	Corneal endothelium
CEpi	Corneal epithelium
CHD	Chromodomain-helicase-DNA binding
CircRNA	Circular RNA
CpG	(Cytosine-Guanine) dinucleotides
CGI	CpG island
CNV	Corneal neovascularization
Col	Collagen
CSt	Corneal stroma
CSSC	Corneal stromal stem cells
DM	Descemet’s membrane
DNMT	DNA methyltransferase
ECM	Extracellular matrix
EpiMT	Epithelial–mesenchymal transition
EV	Extracellular vesicle
FDA	Food and Drug Administration
FECD	Fuchs’ Endothelial Corneal Dystrophy
GCD2	Granular corneal dystrophy type 2
HAT	Histone acetyltransferase
HDAC	Histone deacetylase
HDM	Histone demethylase
HMT	Histone methyltransferase
IFNγ	Interferon-γ
ISWI	Imitation switch
JAK-STAT	Janus kinase/signal transducer and activator of transcription
JNK	c-Jun N-terminal kinase
LncRNA	Long non-coding RNA
LNP	Lipid nanoparticle
MAPK	Mitogen-activated protein kinase
MDS	Myelodysplastic syndromes
miRNA	MicroRNA
myoF	Myofibroblast
NaB	Sodium butyrate
ncRNA	Non-coding RNA
PDGF	Platelet-derived growth factor
RISC	RNA-induced silencing complex
SAHA	Suberoylanilide Hydroxamic Acid
SAM	S-adenosylmethionine
αSMA	α-Smooth muscle actin
SWI/SNF	Switch/sucrose-non-fermenting
TGFβ	Transforming growth factor-β
TSA	Trichostatin A
TNFα	Tumor necrosis factor-α
VEGF	Vascular endothelial growth factor

## References

[1] Sridhar M.S. (2018). Anatomy of cornea and ocular surface. Indian J. Ophthalmol..

[2] Saghizadeh M., Kramerov A.A., Svendsen C.N., Ljubimov A.V. (2017). Concise Review: Stem Cells for Corneal Wound Healing. Stem Cells.

[3] Swamynathan S.K., Swamynathan S. (2023). Corneal epithelial development and homeostasis. Differentiation.

[4] Yam G.H.F., Riau A.K., Funderburgh M.L., Mehta J.S., Jhanji V. (2020). Keratocyte biology. Exp. Eye Res..

[5] Ljubimov A.V., Saghizadeh M. (2015). Progress in corneal wound healing. Prog. Retin. Eye Res..

[6] Flaxman S.R., Bourne R.R.A., Resnikoff S., Ackland P., Braithwaite T., Cicinelli M.V., Das A., Jonas J.B., Keeffe J., Kempen J.H. (2017). Global causes of blindness and distance vision impairment 1990–2020: A systematic review and meta-analysis. Lancet Glob. Health.

[7] Wang E.Y., Kong X., Wolle M., Gasquet N., Ssekasanvu J., Mariotti S.P., Bourne R., Taylor H., Resnikoff S., West S. (2023). Global trends in blindness and vision impairment resulting from corneal opacity 1984–2020: A meta-analysis. Ophthalmology.

[8] Chandran C., Santra M., Rubin E., Geary M.L., Yam G.H. (2024). Regenerative Therapy for Corneal Scarring Disorders. Biomedicines.

[9] West-Mays J.A., Dwivedi D.J. (2006). The keratocyte: Corneal stromal cell with variable repair phenotypes. Int. J. Biochem. Cell Biol..

[10] Jester J.V., Moller-Pedersen T., Huang J., Sax C.M., Kays W.T., Cavangh H.D., Petroll W.M., Piatigorsky J. (1999). The cellular basis of corneal transparency: Evidence for ‘corneal crystallins’. J. Cell Sci..

[11] Wilson S.E. (2020). Corneal myofibroblasts and fibrosis. Exp. Eye Res..

[12] Kwok S.S., Shih K.C., Bu Y., Lo A.C., Chan T.C., Lai J.S., Jhanji V., Tong L. (2019). Systematic review on therapeutic strategies to minimize corneal stromal scarring after injury. Eye Contact Lens.

[13] Kam K.W., Belin M.W., Young A.L. (2015). Monitoring corneal densities following primary pterygium excision with adjuvant topical mitomycin-C application—An observational study of corneal scar changes. Cornea.

[14] Arranz-Marquez E., Katsanos A., Kozobolis V.P., Konstas A.G.P., Teus M.A. (2019). A Critical Overview of the Biological Effects of Mitomycin C Application on the Cornea Following Refractive Surgery. Adv. Ther..

[15] Gain P., Jullienne R., He Z., Aldossary M., Acquart S., Cognasse F., Thuret G. (2016). Global survey of corneal transplantation and eye banking. JAMA Ophthalmol..

[16] Yu T., Rajendran V., Griffith M., Forrester J.V., Kuffova L. (2016). High-risk corneal allografts: A therapeutic challenge. World J. Transpl..

[17] Sampaio L.P., Hilgert G.S.L., Shiju T.M., Santhiago M.R., Wilson S.E. (2022). Topical Losartan and Corticosteroid Additively Inhibit Corneal Stromal Myofibroblast Generation and Scarring Fibrosis After Alkali Burn Injury. Transl. Vis. Sci. Technol..

[18] Pereira-Souza A.L., Ambrosio R., Bandeira F., Salomao M.Q., Souza Lima A., Wilson S.E. (2022). Topical Losartan for Treating Corneal Fibrosis (Haze): First Clinical Experience. J. Refract. Surg..

[19] Du Y., Carlson E.C., Funderburgh M.L., Birk D.E., Pearlman E., Guo N., Kao W.W., Funderburgh J.L. (2009). Stem cell therapy restores transparency to defective murine corneas. Stem Cells.

[20] Basu S., Hertsenberg A.J., Funderburgh M.L., Burrow M.K., Mann M.M., Du Y., Lathrop K.L., Syed-Picard F.N., Adams S.M., Birk D.E. (2014). Human limbal biopsy-derived stromal stem cells prevent corneal scarring. Sci. Transl. Med..

[21] Morgan S.R., Dooley E.P., Kamma-Lorger C., Funderburgh J.L., Funderburgh M.L., Meek K.M. (2016). Early wound healing of laser in situ keratomileusis-like flaps after treatment with human corneal stromal stem cells. J. Cataract Refract. Surg..

[22] Hertsenberg A.J., Shojaati G., Funderburgh M.L., Mann M.M., Du Y., Funderburgh J.L. (2017). Corneal stromal stem cells reduce corneal scarring by mediating neutrophil infiltration after wounding. PLoS ONE.

[23] Ghoubay D., Borderie M., Grieve K., Martos R., Bocheux R., Nguyen T.M., Callard P., Chedotal A., Borderie V.M. (2020). Corneal stromal stem cells restore transparency after N_2_ injury in mice. Stem Cells Transl. Med..

[24] Khandaker I., Funderburgh M.L., Geary M.L., Funderburgh M.L., Jhanji V., Du Y., Yam G.H. (2020). A novel transgenic mouse model for corneal scar visualization. Exp. Eye Res..

[25] Jhanji V., Santra M., Riau A.K., Geary M.L., Yang T., Rubin E., Yusoff N., Dhaliwal D.K., Mehta J.S., Yam G.H. (2022). Combined Therapy Using Human Corneal Stromal Stem Cells and Quiescent Keratocytes to Prevent Corneal Scarring after Injury. Int. J. Mol. Sci..

[26] Shojaati G., Khandaker I., Funderburgh M.L., Mann M.M., Basu R., Stolz D.B., Geary M.L., Dos Santos A., Deng S.X., Funderburgh J.L. (2019). Mesenchymal Stem Cells Reduce Corneal Fibrosis and Inflammation via Extracellular Vesicle-Mediated Delivery of miRNA. Stem Cells Transl. Med..

[27] Yam G.H., Yang T., Geary M.L., Santra M., Funderburgh M.L., Rubin E., Du Y., Sahel J.A., Jhanji V., Funderburgh J.L. (2023). Human corneal stromal stem cells express anti-fibrotic microRNA-29a and 381-5p—A robust cell selection tool for stem cell therapy of corneal scarring. J. Adv. Res..

[28] Santra M., Geary M.L., Coelho J.T., Ali S.R., Chandran C., Dhaliwal D.K., Jhanji V., Yam G.H. (2026). Corneal stromal stem cell-derived extracellular vesicles inhibit corneal neovascularization. Indian J. Ophthalmol..

[29] Tu G.C., Ghalibafan S., Abedi F., Joslin C.E., Hematti P., Djalilian A.R. (2025). Clinical evidence and critical review of mesenchymal stromal cells for corneal and ocular surface diseases. Stem Cells.

[30] Namli I., Gupta D., Singh Y.P., Datta P., Rizwan M., Baykara M., Ozbolat I.T. (2026). Progressive Insights into 3D Bioprinting for Corneal Tissue Restoration. Adv. Healthc. Mater..

[31] Zhang B., Opo F., Nahra D., Chodosh J., Gonzalez-Andrades M., Islam M.M., Patra H.K. (2026). Revolutionizing vision using corneal 3D printing. Trends Biotechnol..

[32] Chen Y., Hong T., Wang S., Mo J., Tian T., Zhou X. (2017). Epigenetic modification of nucleic acids: From basic studies to medical applications. Chem. Soc. Rev..

[33] Ghosh P., Saadat A. (2023). Neurodegeneration and epigenetics: A review. Neurologia.

[34] Esteller M., Dawson M.A., Kadoch C., Rassool F.V., Jones P.A., Baylin S.B. (2024). The Epigenetic Hallmarks of Cancer. Cancer Discov..

[35] Yu X., Zhao H., Wang R., Chen Y., Ouyang X., Li W., Sun Y., Peng A. (2024). Cancer epigenetics: From laboratory studies and clinical trials to precision medicine. Cell Death Discov..

[36] Liang J., Wan C. (2026). Chromatin remodeling and epigenetic regulation in chronic kidney disease. Front. Genet..

[37] Yao W., Hu X., Wang X. (2024). Crossing epigenetic frontiers: The intersection of novel histone modifications and diseases. Signal Transduct. Target. Ther..

[38] Kumar S., Gonzalez E.A., Rameshwar P., Etchegaray J.P. (2020). Non-Coding RNAs as Mediators of Epigenetic Changes in Malignancies. Cancers.

[39] De Riso G., Fiorillo D.F.G., Fierro A., Cuomo M., Chiariotti L., Miele G., Cocozza S. (2020). Modeling DNA Methylation Profiles through a Dynamic Equilibrium between Methylation and Demethylation. Biomolecules.

[40] Dai X., Ren T., Zhang Y., Nan N. (2021). Methylation multiplicity and its clinical values in cancer. Expert Rev. Mol. Med..

[41] Chiang P.K., Gordon R.K., Tal J., Zeng G.C., Doctor B.P., Pardhasaradhi K., McCann P.P. (1996). S-Adenosylmethionine and methylation. FASEB J..

[42] Jiang J., Yan T., Guo F. (2021). Global DNA 5hmC and CK19(5hmC+) Contents: A Promising Biomarker for Predicting Prognosis in Small Hepatocellular Carcinoma. Curr. Oncol..

[43] Hahn M.A., Szabo P.E., Pfeifer G.P. (2014). 5-Hydroxymethylcytosine: A stable or transient DNA modification?. Genomics.

[44] Yang B., Wang J., Tan Y., Yuan R., Chen Z., Zou C. (2021). RNA methylation and cancer treatment. Pharmacol. Res..

[45] Zhao B.S., Roundtree I.A., He C. (2018). Post-transcriptional gene regulation by mRNA modifications. Nat. Rev. Mol. Cell Biol..

[46] Hu J., Lin Y. (2020). Fusarium infection alters the m(6)A-modified transcript landscape in the cornea. Exp. Eye Res..

[47] Klose R.J., Bird A.P. (2006). Genomic DNA methylation: The mark and its mediators. Trends Biochem. Sci..

[48] Bechtel W., McGoohan S., Ziesberg E.M., Muller G.A., Kalbacher H., Salant D.J., Muller C.A., Kalluri R., Zeisberg M. (2010). Methylation determines fibroblast activation and fibrogenesis in the kidney. Nat. Med..

[49] Zhang X., Hu M., Lyu X., Li C., Thannickal V.J., Sanders Y.Y. (2017). DNA methylation regulated gene expression in organ fibrosis. Biochim. Biophys. Acta Mol. Basis Dis..

[50] Li J., Du S., Shi Y., Han J., Niu Z., Wei L., Yang P., Chen L., Tian H., Gao L. (2021). Rapamycin ameliorates corneal injury after alkali burn through methylation modification in mouse TSC1 and mTOR genes. Exp. Eye Res..

[51] Migliaccio G., Morikka J., Del Giudice G., Vaani M., Mobus L., Serra A., Federico A., Greco D. (2024). Methylation and transcriptomic profiling reveals short term and long term regulatory responses in polarized macrophages. Comput. Struct. Biotechnol. J..

[52] Wang X.C., Song K., Tu B., Sun H., Zhou Y., Xu S.S., Lu D., Sha J.M., Tao H. (2023). New aspects of the epigenetic regulation of EMT related to pulmonary fibrosis. Eur. J. Pharmacol..

[53] Ong Tone S., Kocaba V., Bohm M., Wylegala A., White T.L., Jurkunas U.V. (2021). Fuchs endothelial corneal dystrophy: The vicious cycle of Fuchs pathogenesis. Prog. Retin. Eye Res..

[54] Khuc E., Bainer R., Wolf M., Clay S.M., Weisenberger D.J., Kemmer J., Weaver V.M., Hwang D.G., Chan M.F. (2017). Comprehensive characterization of DNA methylation changes in Fuchs endothelial corneal dystrophy. PLoS ONE.

[55] Pan P., Weisenberger D.J., Zheng S., Wolf M., Hwang D.G., Rose-Nussbaumer J.R., Jurkunas U.V., Chan M.F. (2020). Aberrant DNA methylation of miRNAs in Fuchs endothelial corneal dystrophy. Sci. Rep..

[56] Xue T., Qiu X., Liu H., Gan C., Tan Z., Xie Y., Wang Y., Ye T. (2021). Epigenetic regulation in fibrosis progress. Pharmacol. Res..

[57] Huang J., Qin J., Zhu Y., Shen A. (2025). The role of epigenetics in pulmonary fibrosis: Recent advances in mechanistic insights and therapeutic implications. Front. Mol. Biosci..

[58] Perez M., Gomez M., Castellar-Lopez J., Araos P., Mendoza-Torres E., Bolivar S. (2025). Epigenetic modifications in cardiac fibrosis: Recent evidence of new pharmacological targets. Front. Mol. Biosci..

[59] Stewart D.J., Issa J., Kurzrock R., Nunez M.I., Jelinek J., Hong D., Oki Y., Guo Z., Gupta S., Wistuba I.I. (2009). Decitabine effect on tumor global DNA methylation and other parameters in a phase I trial in refractory solid tumors and lymphomas. Clin. Cancer Res..

[60] Buocikova V., Tyciakova S., Pilalis E., Mastrokalou C., Urbanova M., Matuskova M., Demkova L., Medova V., Longhin E.M., Runden-Pran E. (2022). Decitabine-induced DNA methylation-mediated transcriptomic reprogramming in human breast cancer cell lines; the impact of DCK overexpression. Front. Pharmacol..

[61] Alpoim-Moreira J., Szostek-Mioduchowska A., Slyszewska M., Rebordao M.R., Skarzynski D.J., Ferreira-Dias G. (2023). 5-Aza-2′-Deoxycytidine (5-Aza-dC, Decitabine) Inhibits Collagen Type I and III Expression in TGF-β1-Treated Equine Endometrial Fibroblasts. Animals.

[62] Lyu S.Y., Xiao W., Cui G., Yu C., Liu H., Lyu M., Kuang Q.Y., Xiao E.H., Luo Y.H. (2023). Role and mechanism of DNA methylation and its inhibitors in hepatic fibrosis. Front. Genet..

[63] Yonemura H., Futakuchi A., Inoue-Mochita M., Fujimoto T., Takahashi E., Tanihara H., Inoue T. (2019). DNA methyltransferase inhibitor suppresses fibrogenetic changes in human conjunctival fibroblasts. Mol. Vis..

[64] Asada K., Kaji K., Sato S., Seki K., Shimozato N., Kawaratani H., Takaya H., Sawada Y., Nakanishi K., Furukawa M. (2020). Hydralazine Sensitizes to the Antifibrotic Effect of 5-Aza-2′-deoxycytidine in Hepatic Stellate Cells. Biology.

[65] Saba H.I. (2007). Decitabine in the treatment of myelodysplastic syndromes. Ther. Clin. Risk Manag..

[66] Bilokapic S., Strauss M., Halic M. (2018). Histone octamer rearranges to adapt to DNA unwrapping. Nat. Struct. Mol. Biol..

[67] Zhang Y., Sun Z., Jia J., Du T., Zhang N., Tang Y., Fang Y., Fang D. (2021). Overview of Histone Modification. Histone Mutations and Cancer.

[68] Dimitrova E., Turberfield A.H., Klose R.J. (2015). Histone demethylases in chromatin biology and beyond. EMBO Rep..

[69] Oss-Ronen L., Sarusi T., Cohen I. (2022). Histone Mono-Ubiquitination in Transcriptional Regulation and Its Mark on Life: Emerging Roles in Tissue Development and Disease. Cells.

[70] Seto E., Yoshida M. (2014). Erasers of histone acetylation: The histone deacetylase enzymes. Cold Spring Harb. Perspect. Biol..

[71] Okudela K., Mitsui H., Suzuki T., Woo T., Tateishi Y., Umeda S., Saito Y., Tajiri M., Masuda M., Ohashi K. (2014). Expression of HDAC9 in lung cancer—Potential role in lung carcinogenesis. Int. J. Clin. Exp. Pathol..

[72] Chen F., Gao Q., Zhang L., Ding Y., Wang H., Cao W. (2021). Inhibiting HDAC3 (Histone Deacetylase 3) Aberration and the Resultant Nrf2 (Nuclear Factor Erythroid-Derived 2-Related Factor-2) Repression Mitigates Pulmonary Fibrosis. Hypertension.

[73] Wang M., Liao J., Wang J., Xu M., Cheng Y., Wei L., Huang A. (2024). HDAC2 promotes autophagy-associated HCC malignant progression by transcriptionally activating LAPTM4B. Cell Death Dis..

[74] Ganatra D.A., Rajkumar S., Patel A.R., Gajjar D.U., Johar K., Arora A.I., Kayastha F.B., Vasavada A.R. (2015). Association of histone acetylation at the ACTA2 promoter region with epithelial mesenchymal transition of lens epithelial cells. Eye.

[75] Maeng Y.S., Lee G.H., Choi S.I., Kim K.S., Kim E.K. (2015). Histone methylation levels correlate with TGFBIp and extracellular matrix gene expression in normal and granular corneal dystrophy type 2 corneal fibroblasts. BMC Med. Genom..

[76] Lin Y., Wang J., Zhang S., Xu T., Ye J., Reinach P.S., Yan D. (2026). Histone Acetylation Landscape and the Role of HAT1 in Regulating Corneal Epithelial Wound Healing. Investig. Ophthalmol. Vis. Sci..

[77] Zhao B., Chen Y.G. (2014). Regulation of TGF-β Signal Transduction. Scientifica.

[78] Jones D.L., Haak A.J., Caporarello N., Choi K.M., Ye Z., Yan H., Varelas X., Ordog T., Ligresti G., Tschumperlin D.J. (2019). TGFβ-induced fibroblast activation requires persistent and targeted HDAC-mediated gene repression. J. Cell Sci..

[79] Eckschlager T., Plch J., Stiborova M., Hrabeta J. (2017). Histone Deacetylase Inhibitors as Anticancer Drugs. Int. J. Mol. Sci..

[80] Jimenez-Uribe A.P., Gomez-Sierra T., Aparicio-Trejo O.E., Orozco-Ibarra M., Pedraza-Chaverri J. (2021). Backstage players of fibrosis: NOX4, mTOR, HDAC, and S1P; companions of TGF-β. Cell. Signal..

[81] Niki T., Rombouts K., De Bleser P., De Smet K., Rogiers V., Schuppan D., Yoshida M., Gabbiani G., Geerts A. (1999). A histone deacetylase inhibitor, trichostatin A, suppresses myofibroblastic differentiation of rat hepatic stellate cells in primary culture. Hepatology.

[82] Rombouts K., Niki T., Greenwel P., Vandermonde A., Wielant A., Hellemans K., De Bleser P., Yoshida M., Schuppan D., Rojkind M. (2002). Trichostatin A, a histone deacetylase inhibitor, suppresses collagen synthesis and prevents TGF-β(1)-induced fibrogenesis in skin fibroblasts. Exp. Cell Res..

[83] Sharma A., Mehan M.M., Sinha S., Cowden J.W., Mohan R.R. (2009). Trichostatin a inhibits corneal haze in vitro and in vivo. Investig. Ophthalmol. Vis. Sci..

[84] Duvic M., Talpur R., Ni X., Zhang C., Hazarika P., Kelly C., Chiao J.H., Reilly J.F., Ricker J.L., Richon V.M. (2007). Phase 2 trial of oral vorinostat (suberoylanilide hydroxamic acid, SAHA) for refractory cutaneous T-cell lymphoma (CTCL). Blood.

[85] Gronkiewicz K.M., Giuliano E.A., Sharma A., Mohan R.R. (2016). Molecular mechanisms of suberoylanilide hydroxamic acid in the inhibition of TGF-β1-mediated canine corneal fibrosis. Vet. Ophthalmol..

[86] Melnyk S., Lu X., Ronderos V., Choudhary V., Johnson M.H., Watsky M.A., Bollag W.B. (2025). Histone Deacetylase Inhibition Enhances AQP3 Levels in Human Corneal Epithelial Cells and Corneal Wound Healing in Normoglycemic and Diabetic Male Mice. Cells.

[87] Lim R.R., Tan A., Liu Y.C., Barathi V.A., Mohan R.R., Mehta J.S., Chaurasia S.S. (2016). ITF2357 transactivates Id3 and regulate TGFbeta/BMP7 signaling pathways to attenuate corneal fibrosis. Sci. Rep..

[88] Sood S., Sinha N.R., Tiwari A., Ruterschmidt E., Tripathi R., Gupta S., Mohan R.R. (2025). Epigenetic Reprogramming via Sodium Butyrate Induces Corneal Myofibroblast Dedifferentiation In Vitro and Inhibits Fibrosis In Vivo. Investig. Ophthalmol. Vis. Sci..

[89] Allis C.D., Jenuwein T. (2016). The molecular hallmarks of epigenetic control. Nat. Rev. Genet..

[90] Becker P.B., Workman J.L. (2013). Nucleosome remodeling and epigenetics. Cold Spring Harb. Perspect. Biol..

[91] Hassan N.M., Painter N., Howlett C.R., Farrell A.W., Di Girolamo N., Lyons J.G., Halliday G.M. (2014). Brm inhibits the proliferative response of keratinocytes and corneal epithelial cells to ultraviolet radiation-induced damage. PLoS ONE.

[92] Dirscherl S.S., Henry J.J., Krebs J.E. (2005). Neural and eye-specific defects associated with loss of the imitation switch (ISWI) chromatin remodeler in Xenopus laevis. Mech. Dev..

[93] He S., Limi S., McGreal R.S., Xie Q., Brennan L.A., Kantorow W.L., Kokavec J., Majumdar R., Hou H., Edelmann W. (2016). Chromatin remodeling enzyme Snf2h regulates embryonic lens differentiation and denucleation. Development.

[94] Sturm R.A., Larsson M. (2009). Genetics of human iris colour and patterns. Pigment Cell Melanoma Res..

[95] Zhu Y., Kang A., Kuai Y., Guo Y., Miao X., Zhu L., Kong M., Li N. (2023). The chromatin remodeling protein BRG1 regulates HSC-myofibroblast differentiation and liver fibrosis. Cell Death Dis..

[96] Sood S., Kumar R., Sinha N.R., Mohan R.R. (2026). ATAC-seq revealing chromatin accessibility and novel motifs linked to corneal fibrosis. Exp. Eye Res..

[97] Chen E., Bohm K., Rosenblatt M., Kang K. (2020). Epigenetic regulation of anterior segment diseases and potential therapeutics. Ocul. Surf..

[98] O’Brien J., Hayder H., Zayed Y., Peng C. (2018). Overview of MicroRNA Biogenesis, Mechanisms of Actions, and Circulation. Front. Endocrinol..

[99] Liu C.H., Sun Y., Li J., Gong Y., Tian K.T., Evans L.P., Morss P.C., Fredrick T.W., Saba N.J., Chen J. (2015). Endothelial microRNA-150 is an intrinsic suppressor of pathologic ocular neovascularization. Proc. Natl. Acad. Sci. USA.

[100] Lin J.B., Moolani H.V., Sene A., Sidhu R., Kell R., Lin J.B., Dong Z., Ban N., Ory D.S., Apte R.S. (2018). Macrophage microRNA-150 promotes pathological angiogenesis as seen in age-related macular degeneration. JCI Insight.

[101] Urbanska K., Stepien P.W., Nowakowska K.N., Stefaniak M., Osial N., Choragiewicz T., Toro M.D., Nowomiejska K., Rejdak R. (2022). The Role of Dysregulated miRNAs in the Pathogenesis, Diagnosis and Treatment of Age-Related Macular Degeneration. Int. J. Mol. Sci..

[102] Benavides-Aguilar J.A., Morales-Rodriguez J.I., Ambriz-Gonzalez H., Ruiz-Manriquez L.M., Banerjee A., Pathak S., Duttaroy A.K., Paul S. (2023). The regulatory role of microRNAs in common eye diseases: A brief review. Front. Genet..

[103] Tong B.D., Xiao M.Y., Zeng J.X., Xiong W. (2015). MiRNA-21 promotes fibrosis in orbital fibroblasts from thyroid-associated ophthalmopathy. Mol. Vis..

[104] Matthaei M., Hu J., Kallay L., Eberhard C.G., Cursiefen C., Qian J., Lackner E.M., Jun A.S. (2014). Endothelial cell microRNA expression in human late-onset Fuchs’ dystrophy. Investig. Ophthalmol. Vis. Sci..

[105] Li N., Cui J., Duan X., Chen H., Fan F. (2012). Suppression of type I collagen expression by miR-29b via PI3K, Akt, and Sp1 pathway in human Tenon’s fibroblasts. Investig. Ophthalmol. Vis. Sci..

[106] Funari V.A., Winkler M., Brown J., Dimitrijevich S.D., Ljubimov A.V., Saghizadeh M. (2013). Differentially expressed wound healing-related microRNAs in the human diabetic cornea. PLoS ONE.

[107] Alisi L., Giovannetti F., Armentano M., Lucchino L., Lambiase A., Bruscolini A. (2025). Challenging corneal diseases and microRNA expression: Focus on rare diseases and new therapeutic frontiers. Surv. Ophthalmol..

[108] Verma N., Arora S., Singh A.K., Kumar A. (2025). Extracellular Vesicle-Associated miRNAs in Cornea Health and Disease: Diagnostic Potential and Therapeutic Implications. Targets.

[109] Kwon Y., Kim Y., Eom S., Kim M., Park D., Kim H., Noh K., Lee H., Lee Y.S., Choe J. (2015). MicroRNA-26a/-26b-COX-2-MIP-2 Loop Regulates Allergic Inflammation and Allergic Inflammation-promoted Enhanced Tumorigenic and Metastatic Potential of Cancer Cells. J. Biol. Chem..

[110] Chen X., Xiao W., Chen W., Liu X., Wu M., Bo Q., Luo Y., Ye S., Cao Y., Liu Y. (2017). MicroRNA-26a and -26b inhibit lens fibrosis and cataract by negatively regulating Jagged-1/Notch signaling pathway. Cell Death Differ..

[111] Xiang S., Li J., Zhang Z. (2020). miR-26b inhibits isoproterenol-induced cardiac fibrosis via the Keap1/Nrf2 signaling pathway. Exp. Ther. Med..

[112] Zhang S., Cui R. (2020). The targeted regulation of miR-26a on PTEN-PI3K/AKT signaling pathway in myocardial fibrosis after myocardial infarction. Eur. Rev. Med. Pharmacol. Sci..

[113] Zhang W., Wang Q., Feng Y., Chen X., Yang L., Xu M., Wang X., Li W., Niu X., Gao D. (2020). MicroRNA-26a Protects the Heart Against Hypertension-Induced Myocardial Fibrosis. J. Am. Heart Assoc..

[114] Liang H., Xu C., Pan Z., Zhang Y., Xu Z., Chen Y., Li T., Li X., Liu Y., Huangfu L. (2014). The antifibrotic effects and mechanisms of microRNA-26a action in idiopathic pulmonary fibrosis. Mol. Ther..

[115] Hamada A., Shimoji K., Nakashima T., Yamaguchi K., Sakamoto S., Horimasu Y., Masuda T., Iwamoto H., Hamada H., Guo Y. (2025). Systemic miR-26a deficiency attenuates pulmonary fibrosis via PTEN upregulation and downstream TIMP-1 suppression. Mol. Ther. Nucleic Acids.

[116] Yang S., Cui H., Xie N., Icyuz M., Banerjee S., Antony V.B., Abraham E., Thannickal V.J., Liu G. (2013). miR-145 regulates myofibroblast differentiation and lung fibrosis. FASEB J..

[117] Yang J., Liu Q., Cao S., Xu T., Li X., Zhou D., Pan L., Li C., Huang C., Meng X. (2017). MicroRNA-145 Increases the Apoptosis of Activated Hepatic Stellate Cells Induced by TRAIL through NF-kappaB Signaling Pathway. Front. Pharmacol..

[118] Cui S., Liu Z., Tao B., Fan S., Pu Y., Meng X., Li D., Xia H., Xu L. (2021). miR-145 attenuates cardiac fibrosis through the AKT/GSK-3beta/beta-catenin signaling pathway by directly targeting SOX9 in fibroblasts. J. Cell. Biochem..

[119] Yang Y.L., Huang Y.H. (2025). MicroRNA-29a attenuates inflammation and fibrosis in an animal model of NASH through MCJ inhibition and hippo pathway regulation. Eur. J. Pharmacol..

[120] McDaniel G., Li Y., Driscoll T.P. (2025). miR29a-Loaded Extracellular Vesicles Derived from Human Mesenchymal Stem Cells Inhibit Fibrotic and Inflammatory Signaling. ACS Omega.

[121] Gao Y., Chen Y., Mang Y., Zhang X., Li X., Zhang S. (2025). Cholangiocyte-derived exosomal miR-381-3p promotes hepatic stellate cell activation and cholestatic liver fibrosis via targeting Klf6. Regen. Ther..

[122] Singh P., Li F.J., Dsouza K., Stephens C.T., Zheng H., Kumar A., Dransfield M.T., Antony V.B. (2024). Low dose cadmium exposure regulates miR-381-ANO1 interaction in airway epithelial cells. Sci. Rep..

[123] Li C., Li J., Xue K., Zhang J., Wang C., Zhang Q., Chen X., Gao C., Yu X., Sun L. (2019). MicroRNA-143-3p promotes human cardiac fibrosis via targeting sprouty3 after myocardial infarction. J. Mol. Cell. Cardiol..

[124] Tu H., Chen D., Cai C., Du Q., Liu T., Pan T., Sheng L., Xu Y., Teng T., Tu J. (2020). microRNA-143-3p attenuated development of hepatic fibrosis in autoimmune hepatitis through regulation of TAK1 phosphorylation. J. Cell. Mol. Med..

[125] Han L., Zou Y., Yu C. (2022). Targeting CC chemokine ligand (CCL) 20 by miR-143-5p alleviate lead poisoning-induced renal fibrosis by regulating interstitial fibroblasts excessive proliferation and dysfunction. Bioengineered.

[126] Lan W., Chen S., Tong L. (2015). MicroRNA-215 Regulates Fibroblast Function: Insights from a Human Fibrotic Disease. Cell Cycle.

[127] Huang J., Cao Y., Li X., Yu F., Han X. (2022). E2F1 regulates miR-215-5p to aggravate paraquat-induced pulmonary fibrosis via repressing BMPR2 expression. Toxicol. Res..

[128] Yu G., Mu H., Zhou H., Fang F., Cui Y., Wu Q., Xiong Q., Li H. (2021). MicroRNA-361 suppresses the biological processes of hepatic stellate cells in HBV-relative hepatic fibrosis by NF-kappaB p65. Cells Dev..

[129] Zhao R., Zheng Y., Xu K., Huang L., Ding J., Shang X., Tao X., Xin S., Zheng Q., Qian Y. (2025). A multistage microRNA nanotherapeutic to address fibrosis of bacterial keratitis. Nano Today.

[130] Wang L., Yang G., Liu G., Pan Y. (2021). Identification of lncRNA Signature of Tumor-Infiltrating T Lymphocytes with Potential Implications for Prognosis and Chemotherapy of Head and Neck Squamous Cell Carcinoma. Front. Pharmacol..

[131] Uszczynska-Ratajczak B., Lagarde J., Frankish A., Guigo R., Johnson R. (2018). Towards a complete map of the human long non-coding RNA transcriptome. Nat. Rev. Genet..

[132] Han S.H., Ko J.Y., Kang E.S., Park J.H., Yoo K.H. (2023). Long non-coding RNAs: Key regulators of liver and kidney fibrogenesis. BMB Rep..

[133] Micheletti R., Plaisance I., Abraham B.J., Sarre A., Ting C.C., Alexanian M., Maric D., Maison D., Nemir M., Young R.A. (2017). The long noncoding RNA Wisper controls cardiac fibrosis and remodeling. Sci. Transl. Med..

[134] Ilieva M., Uchida S. (2022). Long Non-Coding RNAs in Cardiac and Pulmonary Fibroblasts and Fibrosis. Noncoding RNA.

[135] Zhang L., Gao J., Gong A., Dong Y., Hao X., Wang X., Zheng J., Ma W., Song Y., Zhang J. (2022). The Long Noncoding RNA LINC00963 Inhibits Corneal Fibrosis Scar Formation by Targeting miR-143-3p. DNA Cell Biol..

[136] Bai Y.H., Lv Y., Wang W.Q., Sun G.L., Zhang H.H. (2018). LncRNA NEAT1 promotes inflammatory response and induces corneal neovascularization. J. Mol. Endocrinol..

[137] Verduci L., Tarcitano E., Strano S., Yarden Y., Blandino G. (2021). CircRNAs: Role in human diseases and potential use as biomarkers. Cell Death Dis..

[138] Zhang X.O., Wang H., Zhang Y., Lu X., Chen L.L., Yang L. (2014). Complementary sequence-mediated exon circularization. Cell.

[139] Zhou Y.F., Shi L., Yao J., Sun Y., Shan K., Jiang Q., Yan B. (2019). Microarray Analysis of circRNA Expression Pattern in Corneal Neovascularization. Cornea.

[140] Wu P., Zhang D., Geng Y., Li R., Zhang Y. (2020). Circular RNA-ZNF609 regulates corneal neovascularization by acting as a sponge of miR-184. Exp. Eye Res..

[141] Li Q., Zhang D., Wang Y., Sun P., Hou X., Larner J., Xiong W., Mi J. (2013). MiR-21/Smad 7 signaling determines TGF-β1-induced CAF formation. Sci. Rep..

[142] Ratuszny D., Gras C., Bajor A., Borger A.K., Pielen A., Borgel M., Framme C., Blasczyk R., Figueiredo C. (2015). miR-145 Is a Promising Therapeutic Target to Prevent Cornea Scarring. Hum. Gene Ther..

[143] Men R., Wen M., Zhao M., Dan X., Yang Z., Wu W., Wang M.H., Liu X., Yang L. (2017). MircoRNA-145 promotes activation of hepatic stellate cells via targeting kruppel-like factor 4. Sci. Rep..

[144] Zhang Y., Zhang T., Ma X., Zou J. (2017). Subconjunctival injection of antagomir-21 alleviates corneal neovascularization in a mouse model of alkali-burned cornea. Oncotarget.

[145] Li D., Ji J., Li X., Xie Y., Huang Y., Qin J., Ding X., Wang L., Fan Y. (2025). LNP-encapsulated miRNA29b for corneal repair: A novel approach to combat fibrosis. Mater. Today Bio.

[146] Lempiainen J.K., Garcia B.A. (2023). Characterizing crosstalk in epigenetic signaling to understand disease physiology. Biochem. J..

[147] Du J., Johnson L.M., Jacobsen S.E., Patel D.J. (2015). DNA methylation pathways and their crosstalk with histone methylation. Nat. Rev. Mol. Cell Biol..

[148] Kendall R.T., Feghali-Bostwick C.A. (2014). Fibroblasts in fibrosis: Novel roles and mediators. Front. Pharmacol..

[149] Sanders Y.Y., Tollefsbol T.O., Varisco B.M., Hagood J.S. (2011). Epigenetic regulation of thy-1 by histone deacetylase inhibitor in rat lung fibroblasts. Am. J. Respir. Cell Mol. Biol..

[150] Arzenani M.K., Zade A.E., Ming Y., Vijverberg S.J., Zhang Z., Khan Z., Sadique S., Kallenbach L., Hu L., Vukojevic V. (2011). Genomic DNA hypomethylation by histone deacetylase inhibition implicates DNMT1 nuclear dynamics. Mol. Cell. Biol..

[151] Pathania A.S. (2023). Crosstalk between Noncoding RNAs and the Epigenetics Machinery in Pediatric Tumors and Their Microenvironment. Cancers.

[152] Gaggi G., Hausman C., Cho S., Badalamenti B.C., Trinh B.Q., Di Ruscio A., Ummarino S. (2025). LncRNAs Ride the Storm of Epigenetic Marks. Genes.

[153] Zhang H., Lan S., Ren D., Chen X., Lin Y., Cao Q., Xu W., Wang J., Sol Reinach P., Yan D. (2025). Epigenetic ALYREF/UHRF1/RHOB Axis in Corneal Wound Healing and Implications for Epithelial Tumorigenesis. Investig. Ophthalmol. Vis. Sci..

[154] Ashraf W., Ibrahim A., Alhosin M., Zaayter L., Ouararhni C., Papin C., Ahmad T., Hamiche A., Mely Y., Bronner C. (2017). The epigenetic integrator UHRF1: On the road to become a universal biomarker for cancer. Oncotarget.

[155] Liu Y., Hrit J.A., Chomiak S., Stransky J.R., Hoffman R., Tiedemann R.L., Wiseman A.K., Kariapper L.S., Dickson B.M., Worden E.J. (2025). DNA hypomethylation promotes UHRF1-and SUV39H1/H2-dependent crosstalk between H3K18ub and H3K9me3 to reinforce heterochromatin states. Mol. Cell.

[156] Prasanth M.I., Sivamuruthi B.S., Cheong C.S., Verma K., Tencomnao T., Brimson J.M., Prasansuklab A. (2024). Role of Epigenetic Modulation in Neurodegenerative Diseases: Implications of Phytochemical Interventions. Antioxidants.

[157] Shah R., Patel T., Freedman J.E. (2018). Circulating Extracellular Vesicles in Human Disease. N. Engl. J. Med..

[158] Pidsley R., Zotenko E., Peters T.J., Lawrence M.G., Risbridger G.P., Molloy P., Van Djik S., Muhlhausler B., Stirzaker C., Clark S.J. (2016). Critical evaluation of the Illumina MethylationEPIC BeadChip microarray for whole-genome DNA methylation profiling. Genome Biol..

[159] Johnson N.D., Wu X., Still C.D., Chu A.T., Petrick A.T., Gerhard G.S., Conneely K.N., DiStefano J.K. (2021). Differential DNA methylation and changing cell-type proportions as fibrotic stage progresses in NAFLD. Clin. Epigenet..

[160] Lu Z.J., Ye J.G., Li J.N., Liang J.B., Zhou M., Hu Q.L., Zhang Q.K., Lin Y.H., Zheng Y.F. (2025). Single-Cell Multiomics Analysis of Early Wound Response Programs in the Mouse Corneal Epithelium. Investig. Ophthalmol. Vis. Sci..

[161] Lu T., Ang C.E., Zhuang X. (2023). Spatially resolved epigenomic profiling of single cells in complex tissues. Cell.

[162] Rajasekar P., Patel J., Clifford R.L. (2021). DNA Methylation of Fibroblast Phenotypes and Contributions to Lung Fibrosis. Cells.

[163] Mailleux A.A., Crestani B. (2022). New insights into methylome alterations and consequences during myofibroblastic differentiation in pulmonary fibrosis. Eur. Respir. J..

[164] Liu Z.Y., Song K., Tu B., Lin L.C., Sun H., Zhou Y., Sha J.M., Yang J.J., Zhang Y., Zhao J.Y. (2023). Glycolytic reprogramming in organ fibrosis: New dynamics of the epigenetic landscape. Free Radic. Biol. Med..

[165] Horn P., Tacke F. (2024). Metabolic reprogramming in liver fibrosis. Cell Metab..

[166] Miguel V., Shaw I.W., Kramann R. (2025). Metabolism at the crossroads of inflammation and fibrosis in chronic kidney disease. Nat. Rev. Nephrol..

[167] Sisto M., Lisi S. (2024). Epigenetic Regulation of EMP/EMT-Dependent Fibrosis. Int. J. Mol. Sci..

[168] Zeybel M., Luli S., Sabater L., Hardy T., Oakley F., Leslie J., Page A., Moran Salvador E., Sharkey V., Tsukamoto H. (2017). A Proof-of-Concept for Epigenetic Therapy of Tissue Fibrosis: Inhibition of Liver Fibrosis Progression by 3-Deazaneplanocin A. Mol. Ther..

[169] Cheng M., Li J.J., Niu X.N., Zhu L., Liu J.Y., Jia P.C., Zhu S., Meng H.W., Lv X.W., Huang C. (2023). BRD4 promotes hepatic stellate cells activation and hepatic fibrosis via mediating P300/H3K27ac/PLK1 axis. Biochem. Pharmacol..

[170] Li X.J., Zhou F., Li Y.J., Xue X.Y., Qu J.R., Fan G.F., Liu J., Sun R., Wu J.Z., Zheng Q. (2023). LncRNA H19-EZH2 interaction promotes liver fibrosis via reprogramming H3K27me3 profiles. Acta Pharmacol. Sin..

[171] Tu C., Qian C., Li S., Lin D.Y., Liu Z.Y., Ouyang W.G., Kang X.L., Chen F., Song S., Cai S.Q. (2025). Targeting the chromatin remodeler BAZ2B mitigates hepatic senescence and MASH fibrosis. Nat. Aging.

[172] Acharya N., Kandel R., Roy P., Warraich I., Singh K.P. (2025). Epigenetic therapeutics attenuate kidney injury and fibrosis by restoring the expression of epigenetically reprogrammed fibrogenic genes and signaling pathways. Eur. J. Pharm. Sci..

[173] Jeon K.I., Kumar A., Callan C.L., DeMagistris M., MacRae S., Nehrke K., Huxlin K.R. (2023). Blocking Mitochondrial Pyruvate Transport Alters Corneal Myofibroblast Phenotype: A New Target for Treating Fibrosis. Investig. Ophthalmol. Vis. Sci..

[174] Jeon K.I., Kumar A., Brookes P.S., Nehrke K., Huxlin K.R. (2024). Manipulating mitochondrial pyruvate carrier function causes metabolic remodeling in corneal myofibroblasts that ameliorates fibrosis. Redox Biol..

[175] Jing Y., Li J., Hao P., Xing S., Li X. (2024). Silencing METTL3 Increases HSP70 Expression and Alleviates Fibrosis in Keratocytes. Investig. Ophthalmol. Vis. Sci..

[176] Ahuja N., Sharma A.R., Baylin S.B. (2016). Epigenetic Therapeutics: A New Weapon in the War Against Cancer. Annu. Rev. Med..

[177] Jabbour E., Giralt S., Kantarjian H., Garcia-Manero G., Jagasia M., Kebriaei P., de Padua L., Shpall E.J., Champlin R., de Lima M. (2009). Low-dose azacitidine after allogeneic stem cell transplantation for acute leukemia. Cancer.

[178] Al-Yacoub N., Fecker L.F., Mobs M., Plotz M., Braun F.K., Sterry W., Eberle J. (2012). Apoptosis induction by SAHA in cutaneous T-cell lymphoma cells is related to downregulation of c-FLIP and enhanced TRAIL signaling. J. Investig. Dermatol..

[179] Dimitriu M.A., Lazar-Contes I., Roszkowski M., Mansuy I.M. (2022). Single-Cell Multiomics Techniques: From Conception to Applications. Front. Cell Dev. Biol..

[180] Preissl S., Gaulton K.J., Ren B. (2023). Characterizing cis-regulatory elements using single-cell epigenomics. Nat. Rev. Genet..

